# Efficacy of probiotic and synbiotic supplementation on the length of hospital stays and risk of postoperative mortality in patients undergoing surgery: an umbrella review of systematic reviews and meta-analyses of randomized clinical trials

**DOI:** 10.1186/s40001-025-03756-0

**Published:** 2026-01-08

**Authors:** Kimia Mazinani, Farhang Hameed Awlqadr, Sanaz Mehrabani, Behzad Ebrahimi, Felicity MacIsaac, Seyed Mojtaba Ghoreishy, Faramarz Jalili, Mohammad Ali Hojjati Kermani, Sajjad Moradi

**Affiliations:** 1 Department of Nutrition and Food Science, Maragheh University of Medical Sciences, Maragheh, Iran; 2 Department of Food Science and Quality Control, Halabja Technical College, Sulaimani Polytechnic University, Kurdistan Region, Iraq; 3 Isfahan Cardiovascular Research Center, Cardiovascular Research Institute, Isfahan University of Medical Sciences, Isfahan, Iran; 4 Faculty of Science, Dalhousie University, Halifax, NS Canada; 5 Department of Clinical Nutrition, School of Public Health, Iran University of Medical Sciences, Tehran, Iran; 6 Student Research Committee, School of Public Health, Iran University of Medical Sciences, Tehran, Iran; 7 School of Health Administration, Faculty of Health, Dalhousie University, Halifax, NS Canada; 8 Clinical Tuberculosis and Epidemiology Research Center, National Research Institute of Tuberculosis and Lung Diseases (NRITLD), Masih Daneshvari Hospital, Shahid Beheshti University of Medical Sciences, Tehran, Iran; 9 Research Center for Evidence-Based Health Management, Maragheh University of Medical Sciences, Maragheh, Iran

**Keywords:** Probiotic, Synbiotic, Length of hospital stay, Postoperative mortality, Surgery, Meta-analyses

## Abstract

**Objective:**

This umbrella review was conducted to assess the certainty and validity of all available meta-analyses for intervention trials regarding the impact of synbiotic and probiotic interventions in hospital and Intensive Care Unit (ICU) stay durations, as well as postoperative mortality risk among patients undergoing surgery.

**Methods:**

A comprehensive systematic search was performed by applying Web of Science, Scopus, PubMed, Embase and Cochrane Library until July 20, 2025. Meta-analyses were used to evaluate the effect of synbiotic and probiotic interventions among hospital and ICU stay durations, as well as the postoperative mortality risk in patients undergoing surgery. Effect sizes of synbiotic and probiotic interventions were recalculated by using a random effects model, and the GRADE tool was used to determine evidence certainty.

**Results:**

Forty-eight clinical trials involving 6,378 participants (intervention = 3151; placebo = 3227) across thirty meta-analyses were included in this study. The findings indicated that probiotic supplementation (vs. placebo) significantly reduced the duration of hospital stay [Weighted Mean Difference (WMD): –1.00 days; 95% CI: –1.37 to –0.64; I^2^ = 63.1%; moderate certainty of evidence; P < 0.001; n = 22] among patients undergoing surgery. Synbiotic supplementation showed even greater efficacy, reducing the length of hospital stay by a larger margin (WMD: –2.57 days; 95% CI: –4.51 to –0.64; I^2^ = 83.2%; moderate certainty of evidence; P = 0.009; n = 19). Moreover, the results suggested that synbiotic supplementation did not affect the length of ICU stay. The results indicated that the risk of postoperative mortality did not significantly change after probiotic or synbiotic supplementation (vs. placebo) among patients undergoing surgery.

**Conclusion:**

The current review supports the efficacy of synbiotic and probiotic supplementation on decreasing the length of hospital stay in patients undergoing surgery. However, it is important to note that 42.3% of included systematic reviews and meta-analyses (SRMAs) were rated as 'critically low' quality using the AMSTAR2 tool, which necessitates cautious interpretation of findings.

**Supplementary Information:**

The online version contains supplementary material available at 10.1186/s40001-025-03756-0.

## Introduction

Historically, the evaluation of post-surgery healing has involved utilization of precise outcome measures, through both immediate and long-lasting monitoring. These factors encompass rates of complications and readmissions, rates of re-operations, mortality and morbidity within 30 days, with the most frequently utilized metric being the length of hospital stay [1, 2]. Extended hospitalization is associated with an increased risk of nosocomial infections, patient deconditioning, and elevated healthcare costs [3]. The mortality rate following surgery exhibits substantial variation depending on factors such as the surgical procedure, patient characteristics, and pre-existing medical conditions. When examining major surgeries in older individuals in the United States, it was shown that the mortality rate after one year was 13.4%, with elective surgeries having a reduced mortality rate of 7.4% compared to 22.3% for nonelective surgeries [4]. Further, the incidence of postoperative sepsis exhibits substantial variation among several surgical settings and the occurrence of emergency abdominal surgeries was found to be significantly elevated, reaching 35% [5].

Antibiotic prophylaxis is commonly used to reduce the likelihood of surgical site infections (SSIs), however although antibiotics are highly effective in reducing infections, their use is not without drawbacks. The emergence of antibiotic resistance is a formidable obstacle, intensifying the problem of effectively treating illnesses overtime. Additionally, the use of antibiotics has been linked to the occurrence of antibiotic-induced diarrhea, which can further hinder a patient's progress in recovering [6, 7]. There is a significant focus on interventional methods, as opposed to using medication, to effectively control the duration of hospitalization and reduce the chances of postoperative mortality in surgical patients. Multiple studies have suggested that administering probiotics or synbiotics to patients before undergoing surgery has the potential to greatly decrease the occurrence of postoperative infections, a substantial contributor to morbidity and mortality [8–11]. Probiotic mechanisms involve the displacement of potentially harmful bacteria through competition and the direct inhibition of microbial growth [12]. Probiotics can modify the acidity level of the intestinal lining, generating bacteriocins that hinder the attachment of harmful bacteria to the intestinal cells and the creation of disease-causing substances, as well as safeguarding against the movement of bacteria across tight junctions [12, 13]. In addition, studies have demonstrated that probiotic bacteria can stimulate the production of anti-inflammatory cytokines [14].

Inconsistent results have been reported by several recent meta-analyses concerning the impact of probiotics and synbiotics on postoperative mortality and hospitalization duration [15–17]. Although some reviews show a notable decrease in hospitalization length and infectious complications, others express concern regarding methodological errors and publication bias [18, 19]. Furthermore, inconsistent evidence quality, frequently brought on by the inclusion of trials with low power or a high risk of bias, restricts the findings' ability to be interpreted and applied broadly. Due to a number of interrelated factors, the clinical effects of probiotic/synbiotic supplementation vary greatly, including strain specificity (e.g., Lactobacillus plantarum, Bifidobacterium breve, Saccharomyces boulardii), dosage (10^9^ to 10^11^ CFU/day), timing (pre-, peri-, or postoperative), and whether the supplement is a probiotic or a synbiotic combination [20, 21]. Such variation at the intervention level leads to inconsistent trial results. Additionally, the majority of clinical trials and meta-analyses in this area concentrate on gastrointestinal or digestive surgeries, such as colorectal, gastric, hepatic, and pancreatic procedures, where the risk of infection and disruption of microbiota is particularly high. More than 70% of the participants in recent meta-analyses had gastrointestinal surgeries, with colorectal surgeries being the majority [21, 22].

A systematic synthesis of published meta-analyses, known as an umbrella review, offers a rigorous approach to evaluating the quality, reliability, and limitations of the available evidence considering discrepancies. This method makes it possible to grade the degree of evidence certainty, identify overlapping trials, and compare inclusion criteria across meta-analyses [23, 24]. Previous meta-analyses on this subject exhibit variability in inclusion criteria (e.g., selection of probiotic strains, timing of administration), definitions of outcomes, and categories of surgeries considered. These discrepancies could be a factor in the observed variability and contradictory findings [25–27].

Therefore, this study will conduct a thorough review of published meta-analyses of Randomized Control Trials (RCTs) evaluating the efficacy of probiotic and synbiotic supplementation in lowering the risk of postoperative mortality, as well as the length of hospital and intensive care unit (ICU) stays, for which at least one published meta-analysis of RCTs was available.

## Methods

This umbrella study was conducted according to the Cochrane Handbook for the explanation of “overviews of reviews” [28] and the ‘Grading of Recommendations Assessment Development and Evaluation’ (GRADE) guideline [29]. The outcomes were also reported employing the ‘Preferred Reporting Items for Overviews of Reviews’ (PRIOR) guideline [30] (Supplementary Table 1). The protocol of the present study was approved with PROSPERO (CRD42024579292).

### Systematic search

A search strategy (Supplementary Table 2) was employed for the comprehensive systematic search in the databases of Web of Science, Scopus, PubMed, Embase and Cochrane Library until July 20, 2025. The search found related systematic reviews and meta-analyses (SRMAs), evaluating the efficacy of oral probiotic and synbiotic supplementation on the duration of hospital stay and the risk of postoperative mortality in patients undergoing surgery. One investigator (SM) was responsible for the initial assessment of the publications found, while other investigators confirmed on what keywords and search terms were appropriate. Reference lists of all entered studies also have been reviewed for any missed literature.

### Eligibility criteria

Inclusion criteria consisted of: (1) SRMAs of randomized trials that were performed on all patients undergoing surgery; (2) intervention based on probiotic and synbiotic supplementation; and (3) demonstrated effect estimates in the form of odds ratios (OR), relative risk (RR), and hazard ratio (HR) stating at least 95% confidence interval (95% CI) or weighted mean difference (WMD) with 95% CIs in hospital and ICU duration and risk of postoperative mortality, respectively (Table 1). When more than one SRMA meets our inclusion criteria, we select the SRMA with a greater number of included trials [31]. We excluded original trials, narrative reviews, systematic reviews without meta-analyses, as well as conference reviews or abstracts. Two researchers (KM and SB) screened the titles/abstracts and full texts to assess eligibility. Any discrepancies were addressed by another researcher (SM).

**Table 1:** PICOS criteria for inclusion and exclusion of the studies

Parameter	Criteria
Population	Patients Undergoing Surgery
Intervention	Probiotic and Synbiotic Supplementation
Comparator	Control group
Outcomes	Length of hospital and ICU stay and risk of postoperative mortality
Study design	Systematic reviews and meta-analyses of randomized controlled trials

### Data extraction

Two researchers (SM and KM) extracted the below data from the SRMAs independently: last name of the first author and publication year, average age of the participants in the intervention and control groups, country, sex, effect size, number of intervention trials entered in the largest and most complete meta-analyses, number of trials documented in comparative meta-analyses that used similar supplementation, as well as the number of individuals in either intervention and control groups. We also extracted additional information from every intervention trial entered in the selected SRMAs: dose and duration of the nutritional interventions and adverse events. When multiple SRMAs examined the same outcome, we selected the most comprehensive meta-analysis based on the highest number of included trials. A detailed cross-reference table showing overlap between included studies across different SRMAs is provided in Supplementary Table 4. We carefully examined study overlap to avoid double-counting of individual RCTs in our synthesis.

### Assessment of methodological quality

Two researchers (KM and MK) evaluated the overall quality of the selected SRMAs by employing ‘A Measurement Tool to Assess Systematic Reviews’ (AMSTAR2) [32]. Furthermore, we assessed the quality of the randomized trials included in selected meta-analyses based on the *Cochrane risk-of-bias* tool [33]. Any discrepancies were resolved through discussion with another researcher (SM).

### Data synthesis

The SRMAs selected consisted of the highest number of trials for risk of each result, namely postoperative infection and antibiotic use duration in patients undergoing surgery. Effect estimates and their 95% CIs were extracted from original studies entered in the SRMA. Considering both within-and between-study heterogeneity, the mean differences (MDs) or relative risks (RRs) with 95% CIs illustrated in the forest plot of every meta-analysis were reassessed, employing a conservative random-effects model [34]. Heterogeneity was evaluated by applying the I^2^ statistics while a chi-square test was conducted for homogeneity (P-heterogeneity > 0.10). Heterogeneity was categorized as low (< 25%), moderate (25–50%), high (50–75%), or very high (> 75%) following established guidelines [35]. For all meta-analyses with significant findings and moderate to high heterogeneity (I^2^ > 50%), 95% prediction intervals were calculated to estimate the range of true effects that can be expected in future studies, accounting for between-study heterogeneity [formula: pooled effect ± t(df) × √ (τ^2^ + SE^2^pooled)]. We generated a funnel plot and conducted Egger's test to assess the likelihood of publication bias [36]. All analyses were performed using Stata version 16.0 (StataCorp, College Station, TX, USA), and results with P < 0.05 were considered significant.

### GRADE rating

The GRADE methodology was applied to evaluate the quality of evidence [37]. Each outcome obtained from intervention trials was initially regarded as high, however can be adjusted up or down based on pre-determined criteria. To interpret the magnitude of effect estimates, WMDs were categorized as follows: 0.0–0.2 = trivial, 0.2–0.6 = small, 0.6–1.2 = moderate, 1.2–2.0 = large, 2.0–4.0 = very large, and ≥ 4.0 = extremely large effects [38, 39].

## Results

**Fig. 1:**
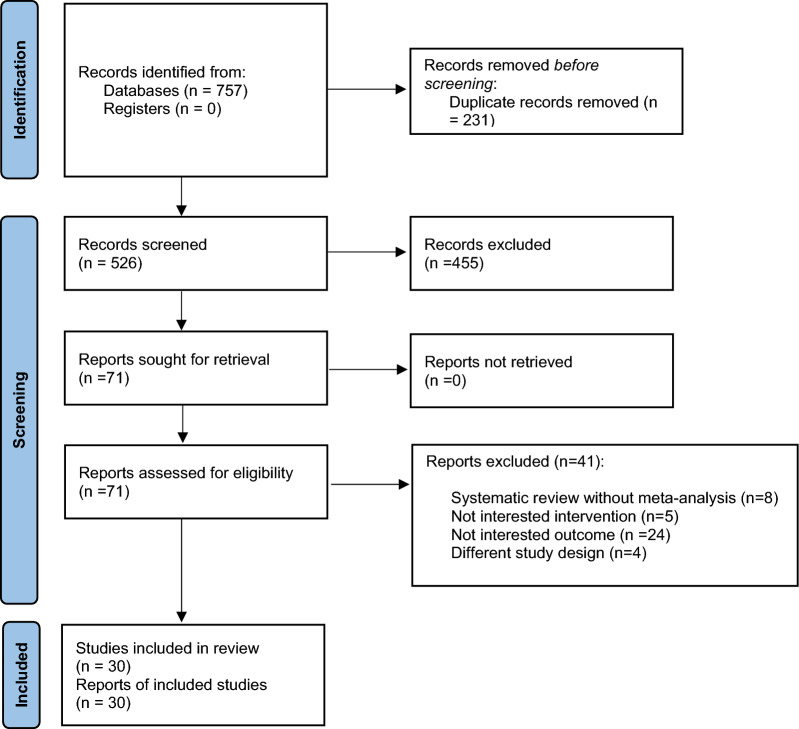
Flow diagram

As illustrated in Fig. 1, a comprehensive systematic search of the specified databases identified 757 records. After omitting duplicates via screening according to the titles/abstracts, 71 studies remained. Next, 41 studies were disregarded for the following reasons: 8 studies were systematic reviews without a meta-analysis, 4 studies had a different design, 24 studies reported unrelated outcomes, and 5 investigated interventions outside the scope of interest. (Supplemental Table 3). Ultimately, 30 SRMAs of RCTs were eligible to be selected in the current study [16, 17, 20, 21, 27, 40–73].

The selected SRMAs included 48 RCTs [8–11, 74–116] with 6378 participants (intervention = 3151, placebo = 3227). Among these RCTs, 38 studies reported the length of hospital stay [11, 75, 77, 79–84, 87–99, 101–116], 9 reported the length of ICU stay [77, 79, 91, 93–95, 100, 101, 115], and 20 reported the risk of postoperative mortality as outcomes [8–11, 74, 76–78, 84–86, 88, 90, 94, 96, 97, 99, 105, 106, 110]. The intervention duration of entered RCTs spanned from 3 to 53 days. The publication dates of the studies ranged from 2002 to 2025. The probiotic interventions ranged from single-strain to multi-strain formulations, with dosages varying from 10^6^ to 10^12^ colony-forming units (CFU) per day. The most used probiotic strains included combinations of Lactobacillus species (L. plantarum, L. acidophilus, L. rhamnosus), Bifidobacterium species (B. longum, B. bifidum), and other beneficial bacteria such as Enterococcus, Streptococcus, Clostridium, and Bacillus species. Prebiotic components in synbiotic formulations primarily consisted of fructooligosaccharides (FOS), galactooligosaccharides (GOS), inulin, pectin, resistant starch, beta-glucan, and oat fiber, with dosages ranging from 2 to 15 g per day. Further, the characteristics of interventions varied considerably across studies, with single-strain probiotics being used in 8 studies (16.7%), while multi-strain formulations were employed in 40 studies (83.3%). The most frequently used individual strains were Lactobacillus plantarum 299v, Lactobacillus acidophilus, Bifidobacterium longum, and Lactobacillus rhamnosus GG. Among synbiotic studies, fructooligosaccharides were the most used prebiotic component (47% of synbiotic studies), followed by galactooligosaccharides (32%) and inulin (28%). The timing of intervention varied, with 15 studies (31%) administering supplements preoperatively only, 12 studies (25%) postoperatively only, and 21 studies (44%) using both pre- and postoperative administration. In addition, the result variables in surgery candidate patients with pancreatic biliary cancers, colorectal cancer, gastric cancer, pancreatic cancers, necrotizing enterocolitis, hepatectomy, liver transplantation, major abdominal surgery biliary cancer surgery, pancreatoduodenectomy, esophageal cancer, extrahepatic bile duct resection, periampullary neoplasms, head and neck cancer, were assessed as well as the adverse events. All reported pooled results were derived from our recalculations rather than relying on the original publications.

### The efficacy of probiotic and synbiotic supplementation on length of hospital stay

**Fig. 2:**
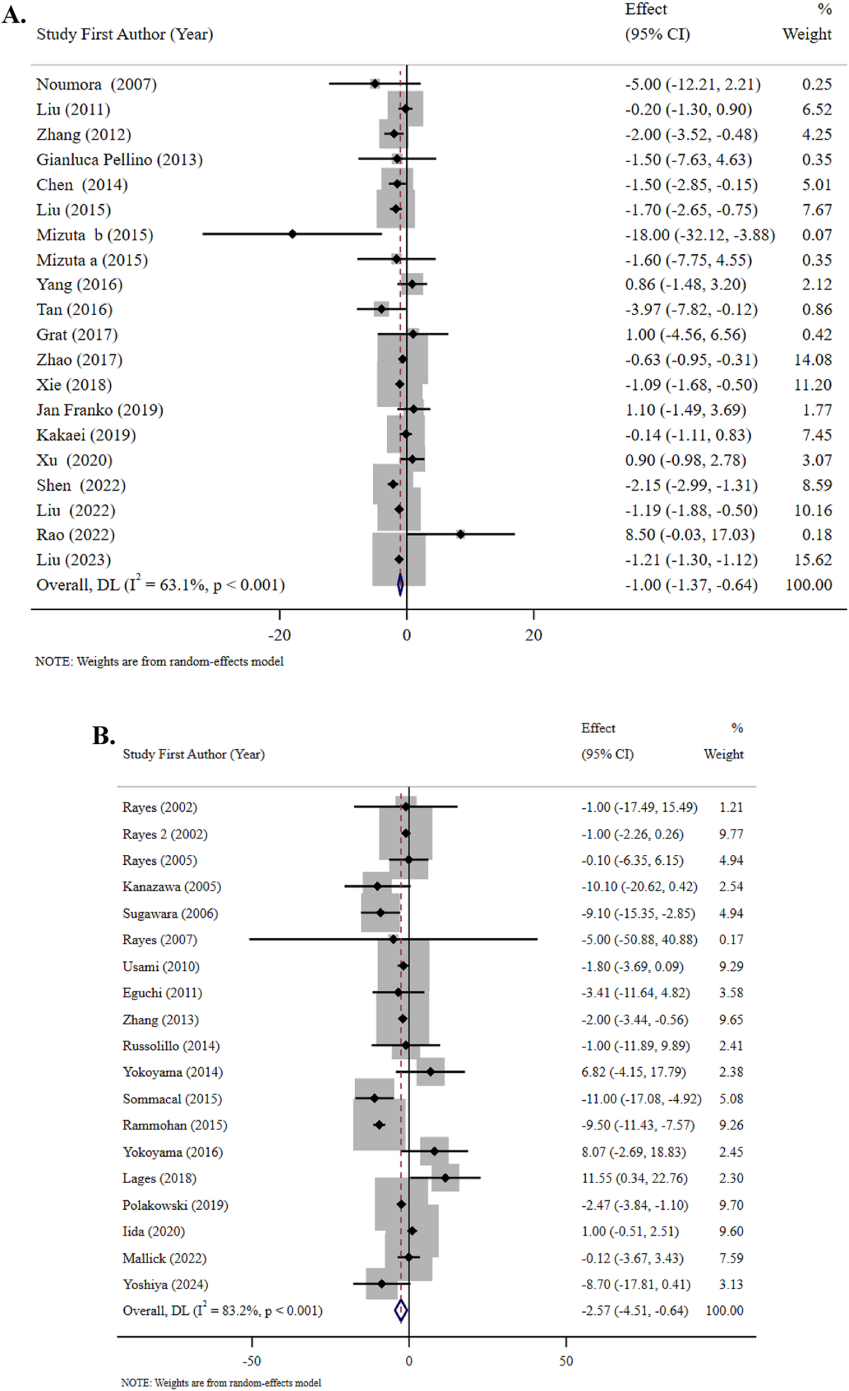

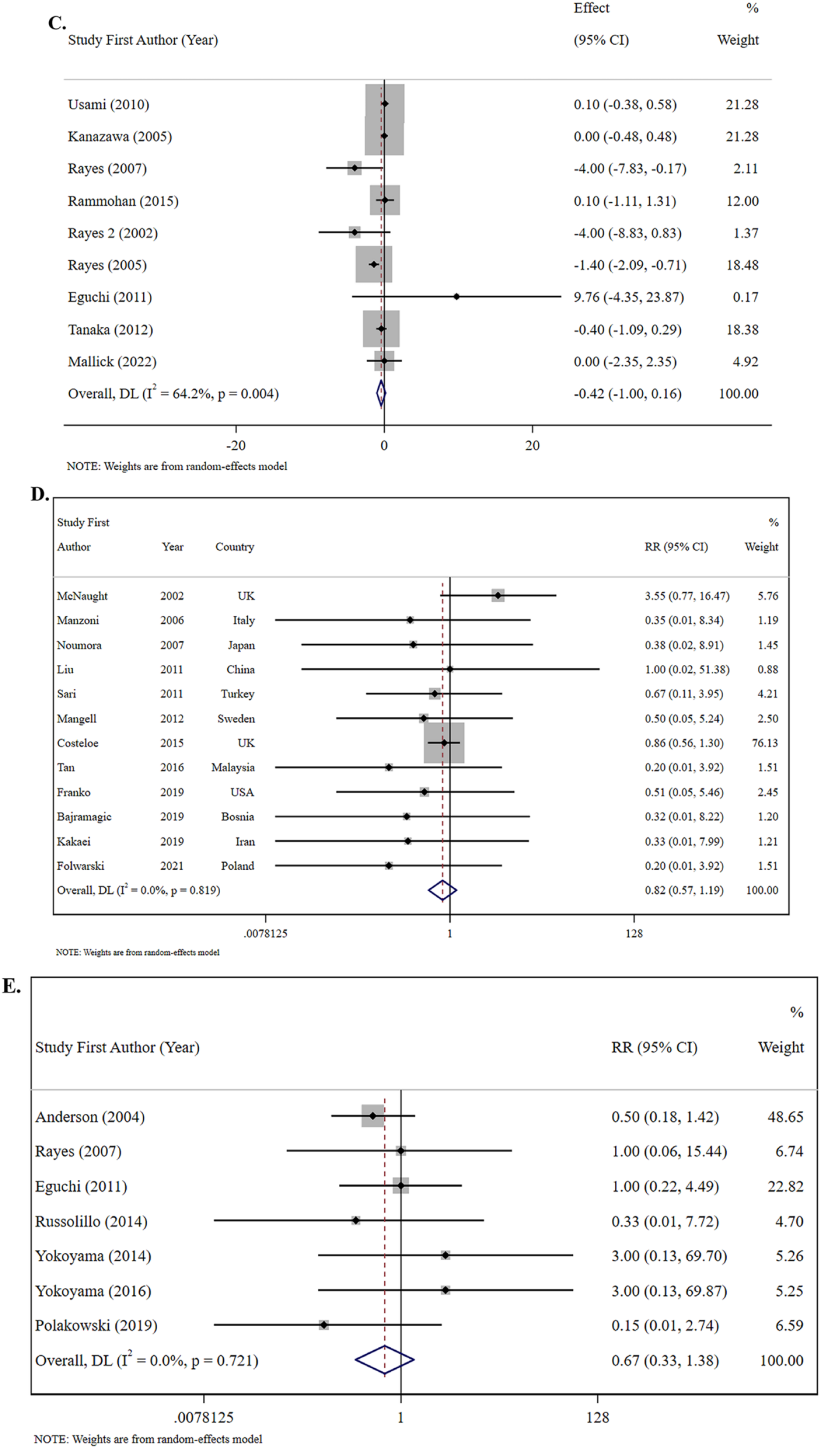
**A** Forest plots demonstrating weighted mean difference and 95% CI for the effects of probiotic supplementation vs. placebo on length of hospital stay. **B** Forest plots demonstrating weighted mean difference and 95% CI for the effects of synbiotic supplementation vs. placebo on length of hospital stay. **C** Forest plots demonstrating weighted mean difference and 95% CI for the effects of synbiotic supplementation vs. placebo on length of ICU stay. **D** Forest plots demonstrating RR and 95% CI of pooled results from the random-effects models to evaluate the effects of probiotic supplementation vs. placebo on risk of postoperative mortality. **E** Forest plots demonstrating RR and 95% CI of pooled results from the random-effects models to evaluate the effects of synbiotic supplementation vs. placebo on risk of postoperative mortality

The outcomes indicated that probiotic supplementation (22 studies, n = 1597 participants) using primarily multi-strain formulations containing combinations of Lactobacillus, Bifidobacterium, Enterococcus, Clostridium, Streptococcus, Bacillus and Lactococcus species at dosages of 10^6^–10^12^ CFU/day for 3–53 days significantly decreased the length of hospital stay (WMD: − 1.00 days, 95% CI: − 1.37, − 0.64, I^2^ = 63.1%; medium evidence certainty; P < 0.001) among patients undergoing surgery. Given the moderate heterogeneity (I^2^ = 63.1%, P < 0.001), the 95% prediction interval was calculated as − 3.26 to 1.26 days, indicating that future studies may show effects ranging from a 3.26-day reduction to a 1.26-day increase in hospital stay **(**Table 2; Fig. 2). This substantial heterogeneity suggests considerable variability in treatment effects across different clinical settings and patient populations, which limits the generalizability of the pooled estimate.

**Table 2:** Efficacy of probiotics and synbiotics supplementation on length of hospital and ICU stay and risk of postoperative mortality in patients undergoing surgery

Intervention	Outcomes	Comparison (s)/intervention	Number of trials	Duration (days)	Strains of probiotics	Kind of prebiotics	**Number of** **Participants** **(Intervention/Control)**	**Effect size** **(95% CI)/[%95 Prediction Interval]**	**Absolute effect (95% CI)**	**P-value**	**I**^**2**^** (%)**	**P **_**heterogeneity**_	**Egger’s test**	**Certainty of** **evidence** **(GRADE)**
Probiotic	Length of hospital stay	Placebo, standard care, fiber and no intervention	22	3–53	Multi-strain probiotics including combinations of Lactobacillus, Bifidobacterium, Enterococcus, Clostridium, Streptococcus, Bacillus and Lactococcus species	–	1597 (796/801)	WMD, −1.00 (−1.37, −0.64)/[−3.26, 1.26]	-	< 0.001	63.1	< 0.001	0.484	Medium
	Risk of postoperative mortality	Placebo, standard care, fiber and no intervention	12	7–53	Multi-strain probiotics including combinations of Lactobacillus, Bifidobacterium, Enterococcus, Clostridium, Streptococcus and Bacillus species	-	2361 (1171/1190)	RR, 0.82 (0.57, 1.19)	RR, −0.24 (−0.62, 0.15)	0.298	0.0	0.819	0.156	Medium
Synbiotic	Length of Hospital Stay	Placebo, standard care and fiber	19	4–30	Multi-strain probiotics including combinations of Lactobacillus, Bifidobacterium, Leuconostoc, Pediacoccus, Clostridium, Streptococcus, and Bacillus species	Fructooligosaccharide, galactooligosaccharides, betaglucan, inulin, pectin, resistant starch, and oat	1358 (610/748)	WMD, −2.57 (−4.51, −0.64)/[−22.34, 17.20]	-	0.009	83.2	< 0.001	0.787	Medium
	Length of ICU Stay	Placebo, standard care and fiber	9	9–25	Multi-strain probiotics including combinations of Lactobacillus, Bifidobacterium, Leuconostoc, Pediacoccus, Clostridium, Streptococcus, and Bacillus species	Fructooligosaccharide, galactooligosaccharides, betaglucan, inulin, pectin, and resistant starch	603 (301/302)	WMD, −0.42 (−1.00, 0.16)/[−3.16, 2.32]	-	0.156	64.2	0.004	0.499	Low
	Risk of Postoperative Mortality	Placebo, standard care and fiber	8	7–21	Multi-strain probiotics including combinations of Lactobacillus, Bifidobacterium, Leuconostoc, Pediacoccus, and Streptococcus species	Fructooligosaccharide, galactooligosaccharides, betaglucan, inulin, pectin, and resistant starch	512 (259/253)	RR, 0.67 (0.33, 1.38)	RR, 0.10 (−0.27, 0.47)	0.278	0.0	0.721	0.537	Medium

*WMD* weighted mean difference, *RR* relative risk

Synbiotic supplementation (19 studies, n = 1358 participants) combining multi-strain probiotics with prebiotics (primarily fructooligosaccharides, galactooligosaccharides, beta-glucan, inulin, pectin, resistant starch, and oat fiber) administered for 4–30 days demonstrated greater efficacy in reducing hospital stay duration (WMD: − 2.57 days, 95% CI: − 4.51, − 0.64, I^2^ = 83.2%; medium evidence certainty; P = 0.009). Substantial heterogeneity (I^2^ = 83.2%, P < 0.001) was reflected in the wide prediction interval (− 22.34 to 17.20 days), suggesting considerable variability in treatment effects across different clinical contexts **(**Table 2; Fig. 2**)**. Substantial heterogeneity (I^2^ = 83.2%, P < 0.001) was reflected in the wide prediction interval (− 22.34 to 17.20 days), suggesting considerable variability in treatment effects across different clinical contexts. This high heterogeneity has important clinical implications, indicating that the effect of synbiotics may vary substantially depending on factors such as surgical type, patient population, specific probiotic strains used, dosage, and timing of administration.

The subgroup analysis revealed significant heterogeneity in treatment effects based on various factors (Table 3). Regarding intervention duration, probiotic supplementation was effective across all durations with no significant between-subgroup differences (P = 0.894): 1–7 days (WD: − 0.96 days, 95% CI: − 1.59, − 0.34, I^2^ = 68.4%; P = 0.002; n = 6 studies), 8–14 days (WD: − 1.13 days, 95% CI: − 1.72, − 0.54, I^2^ = 42.9%; P < 0.001; n = 6 studies), and more than 14 days (WD: − 0.84 days, 95% CI: − 2.17, 0.50, I^2^ = 61.2%; P = 0.220; n = 7 studies). For timing of intervention (P = 0.192 for between-subgroup heterogeneity), preoperative administration showed the largest effect (WD: − 1.64 days, 95% CI: − 2.54, − 0.75, I^2^ = 58.9%; P < 0.001; n = 4 studies), while postoperative administration was not statistically significant (WMD: − 0.57 days, 95% CI: − 1.31, 0.16, I^2^ = 56.5%; P = 0.127; n = 5 studies). Combined pre- and postoperative administration showed moderate efficacy (WMD: − 0.92 days, 95% CI: − 1.61, − 0.23, I^2^ = 53.1%; P = 0.009; n = 10 studies), and strain composition analysis (p = 0.122 for between-subgroup heterogeneity) revealed that single-strain probiotics produced larger effects (WMD: − 2.01 days, 95% CI: − 3.44, − 0.59, I^2^ = 46.4%; P = 0.006; n = 3 studies) compared to multi-strain formulations (WMD: − 0.85 days, 95% CI: − 1.23, − 0.48, I^2^ = 62.9%; P < 0.001; n = 16 studies). In addition, population-based analyses demonstrated significant effects in colorectal cancer patients (WMD: –1.12 days; 95% CI: –1.92 to –0.32; I^2^ = 61.2%; P = 0.006; n = 11 studies) and gastric cancer patients (WMD: –0.94 days; 95% CI: –1.33 to –0.55; I^2^ = 75.7%; P < 0.001; n = 5 studies). No significant effects were observed in liver transplantation (WMD: 1.00 days; 95% CI: –4.56 to 6.56; I^2^ = 0.0%; P = 0.725; n = 1 study) or gastrointestinal surgery (WMD: 8.50 days; 95% CI: –0.03 to 17.03; I^2^ = 0.0%; P = 0.051; n = 1 study). Between-subgroup heterogeneity was not statistically significant (P = 0.160).

**Table 3:** Result of subgroup analysis of included studies in meta-analysis

Intervention	Sub-grouped by	No. of trials	Effect size^a^	95% CI	P-value	I^2^ (%)	P for heterogeneity^b^	P for between subgroup heterogeneity^c^
Probiotic	Length of hospital stay							
	Duration of intervention							0.894
	1–7 days	6	− 0.96	− 1.59, − 0.34	0.002	68.4	0.007	
	8–14 days	6	− 1.13	− 1.72, − 0.54	0.000	42.9	0.119	
	More than 14 days	7	− 0.84	− 2.17, 0.50	0.220	61.2	0.012	
	Time of intervention							0.192
	Preoperative	4	− 1.64	− 2.54, − 0.75	0.000	58.9	0.063	
	Postoperative	5	− 0.57	− 1.31, 0.16	0.127	56.5	0.056	
	Both	10	− 0.92	− 1.61, − 0.23	0.009	53.1	0.019	
	Probiotic strain							0.122
	Single	3	− 2.01	− 3.44, − 0.59	0.006	46.4	0.133	
	Multi	16	− 0.85	− 1.23, − 0.48	0.000	62.9	< 0.001	
	Population type							0.160
	Pancreatic biliary cancers	1	− 5.00	− 12.21, 2.21	0.174	0.0	< 0.001	
	Colorectal cancer	11	− 1.12	− 1.92, − 0.32	0.006	61.2	0.003	
	Gastric cancer	5	− 0.94	− 1.33, − 0.55	0.000	75.7	0.002	
	Liver transplantation	1	1.00	− 4.56, 6.56	0.725	0.0	< 0.001	
	Gastrointestinal surgery	1	8.50	− 0.03, 17.03	0.051	0.0	< 0.001	
	Risk of bias of included studies							0.423
	High	7	− 0.68	− 1.56, 0.20	0.132	61.8	0.011	
	Low	7	− 0.91	− 1.90, 0.08	0.071	68.6	0.004	
	Unclear	5	− 1.21	− 1.30, − 1.12	0.000	0.0	0.843	
	Risk of postoperative mortality							
	Duration of intervention							0.907
	1–7 days	1	0.51	0.05, 5.33	0.574	0.0	< 0.001	
	8–14 days	3	0.99	0.17, 5.72	0.989	47.4	0.149	
	More than 14 days	8	0.79	0.53, 1.17	0.233	0.0	0.960	
	Time of intervention							0.355
	Preoperative	1	0.20	0.01, 3.96	0.291	0.0	< 0.001	
	Postoperative	2	0.25	0.03, 2.29	0.218	0.0	0.839	
	Both	9	0.87	0.60, 1.27	0.470	0.0	0.796	
	Probiotic strain							0.128
	Single	5	0.91	0.61, 1.34	0.618	0.0	0.436	
	Multi	7	0.35	0.11, 1.11	0.076	0.0	0.996	
	Population type							0.580
	Colorectal cancer	7	0.92	0.36, 2.35	0.868	0.0	0.513	
	Pancreatic cancers	2	0.27	0.03, 2.31	0.234	0.0	0.768	
	Enterocolitis necrotizante	3	0.84	0.56, 1.26	0.394	0.0	0.846	
	Type of mortality							0.903
	One year mortality	1	0.32	0.01, 9.17	0.506	0.0	< 0.001	
	30-days mortality	7	0.86	0.34, 2.18	0.757	0.0	0.441	
	In-hospital mortality	3	0.84	0.56, 1.26	0.394	0.0	0.846	
	Not reported	1	0.38	0.02, 8.02	0.534	0.0	< 0.001	
	Risk of bias of included studies							0.877
	High	7	0.88	0.33, 2.29	0.787	0.0	0.429	
	Low	4	0.83	0.55, 1.23	0.347	0.0	0.910	
	Unclear	1	0.38	0.02, 8.02	0.534	0.0	< 0.001	
Synbiotic	Length of ICU stay							
	Duration of intervention							0.017
	8–14 days	2	− 4.00	− 7.00, − 1.00	0.009	0.0	1.000	
	More than 14 days	7	− 0.30	− 0.83, 0.23	0.274	63.3	0.012	
	Time of intervention							0.686
	Postoperative	2	− 1.25	− 4.88, 2.38	0.501	61.6	0.106	
	Both	7	− 0.48	− 1.23, 0.26	0.203	67.5	0.005	
	Probiotic strain							0.543
	Single	2	− 1.37	− 4.51, 1.76	0.390	52.1	0.148	
	Multi	7	− 0.38	− 1.08, 0.33	0.297	69.7	0.003	
	Population type							0.300
	Hepatectomy	2	0.05	− 0.29, 0.39	0.772	0.0	0.773	
	Pancreatic surgery	3	− 0.48	− 1.59, 0.63	0.398	50.4	0.133	
	Liver transplantation	4	− 1.11	− 2.79, 0.58	0.198	37.6	0.186	
	Risk of bias of included studies							0.935
	High	5	− 0.53	− 1.25, 0.19	0.150	70.6	0.009	
	Unclear	4	− 0.45	− 2.32, 1.43	0.642	51.2	0.105	
	Length of hospital stay							
	Duration of intervention							0.261
	1–7 days	3	2.12	− 6.89, 11.14	0.645	63.8	0.063	
	8–14 days	9	− 3.93	− 6.28, − 1.59	0.001	61.8	0.007	
	More than 14 days	7	− 1.02	− 5.26, 3.23	0.638	92.5	< 0.001	
	Time of intervention							0.527
	Preoperative	2	− 5.05	− 11.39, 1.28	0.118	75.7	0.042	
	Postoperative	5	− 1.37	− 3.51, 0.78	0.213	54.6	0.066	
	Both	12	− 2.43	− 5.88, 1.01	0.167	87.8	< 0.001	
	Probiotic strain							0.034
	Single	3	− 0.07	− 1.75, 1.61	0.935	49.8	0.137	
	Multi	16	− 3.17	− 5.49, − 0.86	0.007	80.7	< 0.001	
	Population type							0.003
	Liver transplantation	7	− 0.84	− 2.19, 0.51	0.221	48.7	0.069	
	Major Abdominal surgery	1	− 1.00	− 17.49, 15.49	0.905	0.0	< 0.001	
	Hepatectomy	2	− 4.27	− 11.71, 3.17	0.261	56.8	0.128	
	Biliary cancer	1	− 9.10	− 15.35, − 2.85	0.004	0.0	< 0.001	
	Pancreatoduodenectomy	3	− 1.98	− 17.12, 13.17	0.798	79.9	0.007	
	Oesophageal cancer	1	6.82	− 4.15, 17.79	0.223	0.0	< 0.001	
	Extrahepatic bile duct resection	1	− 1.00	− 11.89, 9.89	0.857	0.0	< 0.001	
	Periampullary neoplasms	1	− 11.00	− 17.08, − 4.92	0.000	0.0	< 0.001	
	Head & neck cancer	1	11.55	0.34, 22.76	0.043	0.0	< 0.001	
	Colorectal cancer	1	− 2.47	− 3.84, − 1.10	0.000	0.0	< 0.001	
	Risk of bias of included studies							0.229
	High	8	− 4.33	− 7.72, − 0.93	0.012	88.6	< 0.001	
	Low	3	− 0.36	− 3.51, 2.78	0.821	54.9	0.109	
	Unclear	8	− 1.51	− 4.52, 1.50	0.326	65.6	0.005	
	Risk of postoperative mortality							
	Duration of intervention							0.152
	7–14 days	4	0.46	0.19, 1.12	0.087	0.0	0.807	
	More than 14 days	3	1.41	0.41, 4.92	0.586	0.0	0.725	
	Time of intervention							0.279
	Preoperative	1	0.15	0.01, 2.48	0.185	0.0	< 0.001	
	Both	6	0.75	0.35, 1.57	0.441	0.0	0.776	
	Population type							0.594
	Colorectal cancer	2	0.43	0.16, 1.14	0.091	0.0	0.430	
	Pancreatoduodenectomy	2	1.62	0.20, 12.96	0.650	0.0	0.608	
	Liver transplantation	1	1.00	0.22, 4.52	1.000	0.0	< 0.001	
	Oesophageal cancer	1	3.00	0.13, 69.46	0.493	0.0	< 0.001	
	Extrahepatic bile duct resection	1	0.33	0.01, 9.17	0.513	0.0	< 0.001	
	Type of mortality							0.678
	30-days mortality	4	0.55	0.23, 1.32	0.180	0.0	0.541	
	90-days mortality	1	0.33	0.01, 9.17	0.513	0.0	< 0.001	
	In-hospital mortality	1	3.00	0.13, 69.55	0.493	0.0	< 0.001	
	Not reported	1	1.00	0.22, 4.52	1.000	0.0	< 0.001	
	Risk of bias of included studies							0.668
	High	1	0.33	0.01, 9.17	0.513	0.0	< 0.001	
	Unclear	6	0.69	0.33, 1.45	0.334	0.0	0.625	

*CI* confidence interval

^a^Calculated by Random-effects model

^b^P heterogeneity within subgroup

^c^P heterogeneity between subgroups

Furthermore, the results of the subgroup analysis for synbiotic supplementation were reported in Table 3. The duration of intervention was shown to significantly affect treatment outcomes (P = 0.261 for between-subgroup heterogeneity). The 8–14 day group showed the most significant benefit (WMD: − 3.93 days, 95% CI: − 6.28, − 1.59, I^2^ = 61.8%; P = 0.001; n = 9 studies), while 1–7 days (WMD: 2.12 days, 95% CI: − 6.89, 11.14, I^2^ = 63.8%; P = 0.645; n = 3 studies) and more than 14 days (WMD: − 1.02 days, 95% CI: − 5.26, 3.23, I^2^ = 92.5%; P = 0.638; n = 7 studies) showed non-significant effects. Strain composition analysis demonstrated significant effect modification between subgroups (P = 0.034), with multi-strain synbiotics demonstrating substantial efficacy (WMD: − 3.17 days, 95% CI: − 5.49, − 0.86, I^2^ = 80.7%; P = 0.007; n = 16 studies), while single-strain formulations showed no significant effect (WMD: − 0.07 days, 95% CI: − 1.75, 1.61, I^2^ = 49.8%; P = 0.935; n = 3 studies). A population-based subgroup analysis showed significant heterogeneity (P = 0.003). Further, specific surgical populations demonstrated varying responses: biliary cancer patients showed the largest reduction (WMD: − 9.10 days, 95% CI: − 15.35, − 2.85, I^2^ = 0.0%; P = 0.004; n = 1 study), as well as periampullary neoplasms (WMD: − 11.00 days, 95% CI: − 17.08, − 4.92, I^2^ = 0.0%; P < 0.001; n = 1 study), and colorectal cancer patients (MD: − 2.47 days, 95% CI: − 3.84, − 1.10, I^2^ = 0.0%; P < 0.001; n = 1 study). Conversely, head and neck cancer patients showed an increased hospital stay (MD: 11.55 days, 95% CI: 0.34, 22.76, I^2^ = 0.0%; P = 0.043; n = 1 study), while liver transplantation patients showed non-significant reduction in their hospitalization duration (MD: − 0.84 days, 95% CI: − 2.19, 0.51, I^2^ = 48.7%; P = 0.221; n = 7 studies).

### The efficacy of synbiotic supplementation on length of ICU stay

Regarding ICU stay outcomes, synbiotic interventions (9 studies, n = 603 participants) utilizing multi-strain probiotics with prebiotic combinations administered for 9–25 days was shown to not significantly reduce ICU length of stay (WMD: − 0.42 days, 95% CI: − 1.00, 0.16, I^2^ = 64.2%; low evidence certainty; P = 0.156). Moderate heterogeneity (I^2^ = 64.2%, P = 0.004) was shown, with a prediction interval of − 3.16 to 2.32 days (Table 2; Fig. 2). The subgroup analysis in Table 3 demonstrated that synbiotic administration was more effective on length of ICU stay among studies in the 8–14-day group (SMD: − 4.00 days, 95% CI: − 7.00, − 1.00, I^2^ = 0.0%; P = 0.009) (vs. more than 14 days).

### The efficacy of probiotic and synbiotic supplementation on the risk of postoperative mortality

**Fig. 3:**
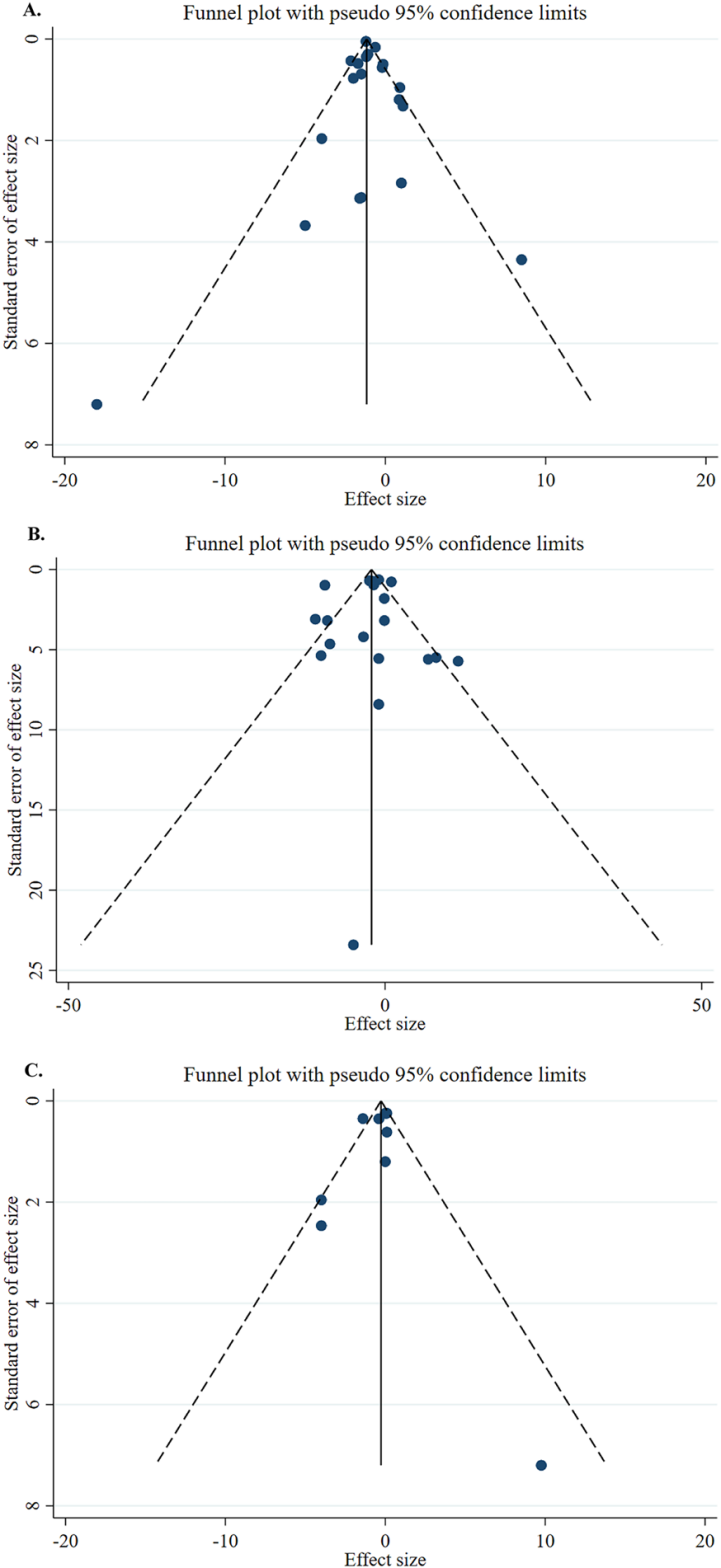

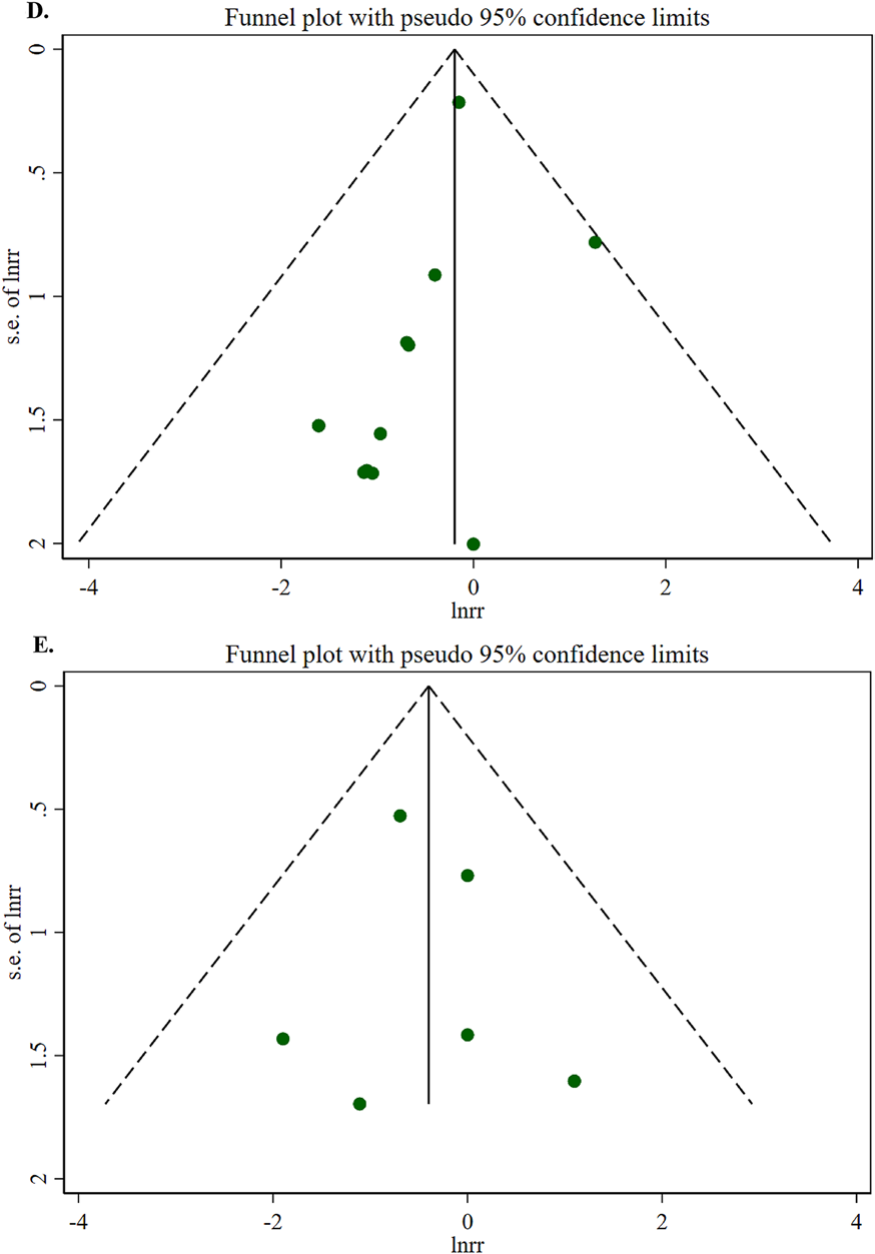
**A** Funnel plots demonstrating weighted mean difference and 95% CI for the effects of probiotic supplementation vs. placebo on length of hospital stay. **B** Funnel plots demonstrating weighted mean difference and 95% CI for the effects of synbiotic supplementation vs. placebo on length of hospital stay. **C** Funnel plots demonstrating weighted mean difference and 95% CI for the effects of synbiotic supplementation vs. placebo on length of ICU stay. **D** Funnel plots demonstrating RR and 95% CI of pooled results from the random-effects models to evaluate the effects of probiotic supplementation vs. placebo on risk of postoperative mortality. **E** Funnel plots demonstrating RR and 95% CI of pooled results from the random-effects models to evaluate the effects of synbiotic supplementation vs. placebo on risk of postoperative mortality

Regarding postoperative mortality risk, neither probiotic supplementation (12 studies, n = 2361 participants, intervention duration 7–53 days) nor synbiotic supplementation (8 studies, n = 512 participants, intervention duration 7–21 days) was shown to significantly affect mortality rates (RR: 0.82, 95% CI: 0.57, 1.19, P = 0.298 and RR: 0.67, 95% CI: 0.33, 1.38, P = 0.278, respectively). (Table 2; Fig. 2). As shown in Table 3, there were no significant differences between the subgroup analyses. As illustrated in Fig. 3, no evidence of publication bias was observed among the included studies. It should be emphasized that postoperative mortality data were limited and imprecise, with wide confidence intervals (RR: 0.82, 95% CI: 0.57, 1.19 for probiotics; RR: 0.67, 95% CI: 0.33, 1.38 for synbiotics) reflecting the small number of events and potential underpowering for this rare outcome.

### Adverse events

Supplementary Table 6 showed adverse events following the administration of probiotics or synbiotics in the primary 48 RCTs that were selected for the umbrella review. Overall, the majority of included trials (32 out of 48 studies, 66.7%) either reported no adverse events or did not report adverse event data. Among the 16 studies (33.3%) that provided specific adverse event information, the interventions were generally well-tolerated with predominantly mild gastrointestinal side effects.

Regarding the probiotic interventions, adverse events were reported in 12 studies with evaluable data from approximately 1156 participants in the intervention groups. The most frequently reported adverse events included diarrhea (pooled incidence: 3.8%, 95% CI: 2.1–6.8%, reported in 8 studies), abdominal cramps/pain (pooled incidence: 2.9%, 95% CI: 1.5–5.7%, reported in 6 studies), and nausea (pooled incidence: 1.7%, 95% CI: 0.8–3.4%, reported in 4 studies). Taste-related issues were reported in 2 studies affecting 21 patients. Notably, Liu et al. [83] and Yang et al. [104] reported a significantly lower incidence of diarrhea in probiotic groups compared with control groups (P < 0.05), suggesting potential protective effects. Severe adverse events were rare, with only Franko et al. [11] reporting 5 out of 11 readmissions in the probiotic group due to dehydration from diet intolerance and/or diarrhea.

Regarding the synbiotic interventions, adverse events were evaluated in 14 studies encompassing approximately 892 participants in the intervention groups. The adverse event profile was similar to the probiotics group, with diarrhea being the most frequent (pooled incidence: 4.2%, 95% CI: 2.3–7.6%, reported in 9 studies), followed by abdominal distention/bloating (pooled incidence: 3.1%, 95% CI: 1.6–6.0%, reported in 7 studies), and abdominal cramps (pooled incidence: 2.7%, 95% CI: 1.3–5.5%, reported in 8 studies). Several studies specifically attributed diarrhea to the prebiotic component, particularly oligofructose. Anderson et al. [74] noted that 4 patients experienced diarrhea related to oligofructose intake, with 1 patient finding the preparation unpalatable. The majority of reported adverse events were transient and resolved with temporary dose reduction or discontinuation.

A comparative analysis revealed no significant difference in overall adverse event rates between probiotic and synbiotic interventions (4.8% vs. 5.3%, P = 0.67). Importantly, no serious adverse events or treatment-related hospitalizations were directly attributed to probiotic or synbiotic supplementation. Studies reporting adverse events showed adequate safety monitoring, with most events being Grade 1 (mild) according to Common Terminology Criteria for Adverse Events (CTCAE) classification. The safety profile appeared consistent across different surgical populations, intervention durations (3–53 days), and dosages (10^6^–10^12^ CFU/day).

### Methodological quality

Outcomes of the quality assessment in the selected SRMAs are displayed in Supplementary Table 7. Evidence of quality in the selected SRMAs are “high”, “low” and “critically low” at 20%, 43.3%, and 36.6%, respectively. The majority of the SRMAs are indicated as “critically low”, evident from the quality of their results and the exclusion of their following the outlined domains: (1) The established method of review before writing was not documented; (2) The risk of bias of each chosen RCT was not reported on the author’s sources of funding.

## Discussion

This umbrella review estimated the effect of probiotic and synbiotic supplementation on hospital stay duration and postoperative mortality in patients undergoing surgery. The results of this study revealed a significant reduction in hospital stay after supplementation, however no significant effect on postoperative mortality was shown. Moreover, synbiotic supplementation was shown to not significantly affect ICU length of stay compared with placebo. Subgroup analyses yielded additional findings, as described below.

The subgroup analysis demonstrated a significant association between probiotic supplementation duration and the length of hospital stay in patients undergoing surgery. Specifically, probiotic supplementation for less than 14 days was determined to be associated with a significant decrease in the length of hospital stay vs. supplementation for more than 14 days. It is imperative to acknowledge that multiple factors, such as the probiotic strains and dosage employed, in studies involving varying durations of intervention may exert an influence on the research outcome. For instance, a meta-analysis study revealed that administration of Lactiplantibacillus plantarum (L. plantarum), whether used independently or in conjunction with other probiotic strains, demonstrated a notable decrease in the risk of overall infections. Conversely, clinical studies that did not use L. plantarum did not show a discernible effect on infectious complications [117]. Additionally, the subgroup analysis showed that a reduction in the length of hospital stay was facilitated by probiotic use during the post and perioperative phases, but not before the surgery. It is plausible that the limited efficacy of preoperative probiotics stems from the insufficient length of time that the supplements were administered for prior to the surgery. A more extended supplementation period may be required to elicit substantial modifications in the gut microbiota or to bolster immune responses effectively. Moreover, the effect of probiotic supplementation prior to surgery on the duration of hospital stay has only been examined in 2 studies. The results show that more research is necessary, especially in instances involving prolonged probiotic administration prior to surgery.

The results of this study demonstrated that the use of probiotic supplements comprising multiple strains, and synbiotics containing several probiotic strains, significantly decreased the duration of hospital stay, but not when only one single strain of probiotics was used. The potential benefits of multi-strain probiotics may be enhanced due to the synergistic and additive interactions that occur among the various individual strains [118]. This cooperative effect leads to an enhanced ability to adhere to mucosal surfaces and a greater capacity to impede pathogens in the gastrointestinal tract [119]. Furthermore, evidence indicates that probiotic supplementation is more effective in reducing hospital stay among patients undergoing colorectal and gastric cancer surgery, whereas no significant effect was observed in pancreatic biliary cancer surgery. A limited number of studies have evaluated the effectiveness of probiotic supplementation on the length of hospital stay for patients with pancreatic biliary cancers, therefore, it appears that further research in this area could yield more definitive conclusions. Additionally, it should be noted that there is a considerable risk of postoperative complications following pancreatic surgical procedures; these can vary in likelihood from 40 to 70% [120–122]. Given the high rate of complications that follow pancreatic surgery, longer-term and higher-dose administration of probiotic supplementation may be necessary.

The subgroup analysis revealed that synbiotic supplementation reduced hospital stay in liver transplantation surgery, biliary cancer, periampullary neoplasms, head and neck cancer, and colorectal cancer, but had no effect on others. It is critical to recognize that several factors can affect how long a patient stays in the hospital, including age, medical conditions, discharge conditions, type of care, ethnicity, physician specialty, and referral source [123, 124]. Given these factors, it is essential to provide a nuanced interpretation of the analysis. Furthermore, the findings of our study indicate that synbiotic supplementation can successfully reduce hospital stay length, particularly when the intervention period lasts between 8 and 14 days, while studies with intervention durations longer than 14 days, or shorter than 8 days, did not show any discernible effect on the length of hospital stay. The brief duration of probiotic intervention was shown to be insufficient in modifying the composition of the gut microbiota required to induce an immune-boosting effect. Additionally, the composition of gut microbiota can change as a result of surgical stress and other perioperative factors [125], indicating that longer intervention times might be necessary to produce more comprehensive results. Moreover, in contrast to studies with low risk of bias, synbiotic supplements in studies with high risk of bias were shown to significantly shorten hospital stays. It is imperative to use caution when interpreting these results due to the differences in outcomes between high-risk and low-risk studies.

The substantial heterogeneity observed across studies (I^2^ ranging from 63.1% to 83.2%) has important clinical implications that warrant emphasis. Our subgroup analyses revealed that heterogeneity was partly explained by differences in intervention characteristics (duration, timing, strain composition) and patient populations (surgical type, underlying conditions). However, residual heterogeneity likely stems from additional factors including variations in probiotic dosages (10^6^–10^12^ CFU/day), administration protocols, baseline microbiome composition, nutritional status, antibiotic use patterns, and other unmeasured confounders. The wide prediction intervals calculated for hospital stay outcomes indicate that the effect of probiotic and synbiotic interventions may differ substantially in specific clinical contexts, limiting the direct applicability of pooled estimates to individual patient care decisions.

The potential influence of probiotic consumption on reducing the duration of hospitalization could be attributed to its impact on immune system functionality. The intestinal microbiome is crucial for the proper functioning and structural integrity of the gastrointestinal tract, as well as for the preservation of immune balance [126]. Probiotic consumption has been demonstrated to encourage the colonization of beneficial commensal microflora in the gastrointestinal system, capable of eliminating harmful microbes directly, thereby reducing infection risk. Furthermore, probiotics compete with pathogens for essential nutrients, limiting their capacity to flourish within the gastrointestinal tract, and are pivotal in fortifying the immune system situated within the gut [127]. Therefore, a change in the microbial community, as well as intestinal immune cell activity, may be responsible for the beneficial effects that probiotics have on immune system functions [128–130]. Probiotics are shown to be critical in immune response modulation by promoting increased antibody production and boosting the activity of natural killer cells and macrophages. As a result, with probiotic administration, the host defense system is strengthened and better able to resist potential infectious agents [131, 132]. Furthermore, when the gut barrier is strengthened by probiotics, there is improvement in the levels of inflammation, the condition known as leaky gut, and the translocation of harmful pathogens from the intestinal tract to other areas of the body [131]. In addition, probiotics generate lactic acid, hydrogen peroxide, and bacteriocins: antimicrobial substances preventing harmful microorganisms from growing [133–135]. While stress can alter the makeup of gut bacteria via pathways mediated by corticotropin, which can increase gut epithelial permeability and trigger an inflammatory response throughout the body [136], probiotics can safeguard the integrity of the intestinal mucosal barrier, reducing damage and permeability of the intestinal mucosa, improving the systemic inflammatory response, and facilitating the restoration of gut microbiota in patients undergoing surgical procedures [136]. Synbiotics and probiotics have a major impact on modulating systemic inflammation [137]. Aside from the known benefits of probiotics, the efficacy of synbiotics may also be attributable to the activity of prebiotics such as fructooligosaccharides (FOS). Prebiotics selectively stimulate the growth and activity of beneficial gastrointestinal bacteria such as Lactobacillus and Bifidobacterium species [138]. One of the important mechanisms by which prebiotics exert their effects is through fermentation by the gut microbiota, leading to the production of short-chain fatty acids (SCFAs) such as acetate, propionate, and butyrate. SCFAs play a significant role in gut health by strengthening the intestinal epithelial barrier [139], regulating local and systemic immune responses [140], and exerting anti-inflammatory effects [141]. These mechanisms can be beneficial in surgical patients who, as a result of surgical stress, develop gut dysbiosis and increased intestinal permeability. Hence, the synergistic action of prebiotic and probiotic combinations in synbiotic formulations may yield greater efficacy than probiotics alone.

There was no effect found of probiotic or synbiotic supplementation on post-surgical mortality in this study. This finding may be due to the multifactorial etiology of mortality, as it is influenced by factors such as underlying diseases, illness severity, quality of care, and immune response-beyond the pathways affected by probiotics, such as gut microbiota modulation and immune enhancement [142–146]. Additionally, the duration, dosage, and particular strains of probiotics used may have had an impact on the study's findings. It is plausible that either the specific probiotic strains utilized did not yield a noteworthy effect on survival rates during the surgical recovery phase, or the duration of administration was too short to do so.

Multiple complex factors extending beyond the gut microbiota–immune pathways modulated by probiotics have been shown to influence postoperative mortality, including patient comorbidities, surgical complexity, anesthetic management, and healthcare system quality. The mortality analysis in this study was limited by the relatively short follow-up periods in most included studies (typically 30 days) and potential underpowering, as mortality is a rare outcome in elective surgery populations. Additionally, the lack of significant effect on mortality should be interpreted in the context of the multifactorial nature of surgical mortality and the limited statistical power to detect differences in this relatively rare outcome. Further research with larger cohorts and extended follow-up, adequately powered for mortality outcomes, is required.

Although evidence supports the potential benefits of probiotic and synbiotic supplementation in surgical patients, it is important to note that these interventions are not safe in all patient populations. Probiotics, in particular, should be used with caution in immunocompromised patients [147], or patients with central venous catheters, as supplementation can lead to probiotic-associated bloodstream infections [148]. Therefore, clinical trials and guidelines in the future are needed to clearly establish the risk–benefit ratio and contraindications. Surgical patient use recommendations must be founded not only on efficacy, but also on patient safety.

While our findings demonstrate statistically significant reductions in hospital stay duration (WMD: − 1.00 days for probiotics, − 2.57 days for synbiotics), the clinical significance requires careful interpretation in light of the substantial heterogeneity observed (I^2^ = 63.1%–83.2%) and the variable quality of included evidence. The 1-day reduction with probiotics, though modest, may translate to meaningful healthcare cost savings and reduced nosocomial infection risk when applied across large surgical populations. The 2.57-day reduction with synbiotic approaches appears more clinically meaningful; however, the wide prediction intervals (− 22.34 to 17.20 days) indicate that treatment effects may vary dramatically across different clinical settings. Furthermore, minimally clinically important differences (MCIDs) for hospital stay duration in surgical patients are not well-defined in the literature, limiting our ability to definitively assess clinical significance.

Consideration of the cost-effectiveness of synbiotic versus probiotic interventions is also necessary; however, none of the included SRMAs reported economic evaluations. Given the potentially greater clinical effects of synbiotics (− 2.57 vs − 1.00 days reduction in hospital stay) but also likely higher costs, cost–benefit studies are essential to determine whether the incremental clinical benefit justifies additional monetary expenses, especially in resource-scarce settings. Such economic analyses should account for not only supplement costs but also potential savings from reduced hospital stays, fewer complications, and decreased antibiotic use.

This study has several strengths, including the use of an umbrella review approach, which synthesizes evidence from multiple meta-analyses to provide a comprehensive overview of the impact of probiotics and synbiotics on mortality and hospitalization duration. Furthermore, we utilized the AMSTAR 2 tool to assess the quality of each study included to differentiate between higher and lower quality evidence. We evaluated the certainty of the evidence in accordance with the GRADE criteria, ensuring transparent and interpretable findings. Collectively, these approaches helped to minimize bias and increase the credibility and generalizability of our findings for policy and clinical practice. However, it is essential to acknowledge the substantial constraints present in this study, as they significantly influence the interpretation of the findings. The most critical limitation is that 42.3% of included SRMAs were rated as "critically low" quality, with an additional 43.3% rated as "low" quality using the AMSTAR2 tool. This means that only 20% of included reviews met high-quality standards. The predominance of low-quality evidence potentially undermines the validity of conclusions drawn overall and mandates particularly cautious interpretation. The primary quality deficiencies included: (1) failure to document an established review protocol before conduct; (2) inadequate reporting of risk of bias in individual randomized controlled trials; and (3) insufficient disclosure of funding sources. These methodological shortcomings in the underlying systematic reviews limit our confidence in the pooled estimates and highlight the urgent need for higher-quality systematic reviews in this field.

Moreover, this study is based on peer-reviewed systematic reviews and meta-analyses that may carry a certain risk of publication bias. We acknowledge the substantial heterogeneity observed in our meta-analyses (I^2^ = 63.1–83.2%). To address this limitation, we conducted comprehensive subgroup analyses examining intervention duration, timing (pre-, post-, or perioperative), strain composition (single vs. multi-strain), and surgical population types. These analyses revealed that heterogeneity was partly explained by differences in intervention characteristics and patient populations. However, residual heterogeneity may stem from variations in probiotic dosages (10^6^–10^12^ CFU/day), administration protocols, and unmeasured confounders across studies. Furthermore, due to the presence of moderate to high heterogeneity (I^2^ = 63.1–83.2%), 95% prediction intervals were calculated to estimate the range of effects expected in future studies. The prediction intervals were shown to be substantially wider than the confidence intervals, indicating considerable between-study variability that limits the generalizability of pooled estimates and suggests that treatment effects may vary significantly across different clinical settings and patient populations. Heterogeneity in probiotic doses and strains among the included studies have been shown to limit effective dose–response relationship analysis. Moreover, heterogeneity between study populations, types of surgery, and quality in SRMAs may also reduce generalizability.

## Conclusion

This umbrella review must be interpreted with significant caution given that 42.3% of included systematic reviews and meta-analyses were rated as "critically low" quality using the AMSTAR2 assessment tool, with only 20% meeting high-quality standards. This predominance of low-quality evidence substantially impacts the robustness and reliability of the findings presented.

With this limitation acknowledged, the findings of this umbrella review indicate that probiotic and synbiotic supplementation is associated with shorter hospital stays in surgical populations (− 1.00 days for probiotics and − 2.57 days for synbiotics). Subgroup analyses showed that the use of multi-strain probiotics or synbiotics, as well as certain surgical procedures, were associated with the greatest benefits from these interventions. No significant decrease was found to occur in postoperative mortality or length of ICU stay. The modest reductions in hospital stay, while statistically significant, should be interpreted in the context of substantial heterogeneity (I^2^ = 63.1%–83.2%), wide prediction intervals, and variable evidence quality across included studies. The clinical significance of a 1-day reduction with probiotics may be meaningful at the population level but requires individualized assessment. The apparently larger effect of synbiotics (2.57-day reduction) is accompanied by particularly high heterogeneity (I^2^ = 83.2%), suggesting highly variable effects across different clinical contexts.

Further, high-quality and properly powered randomized controlled trials with standardized definitions, clear clinical endpoints, and consistent intervention protocols are needed to determine the true efficacy of probiotic and synbiotic supplementation on the length of hospital stay and mortality risk. Future research should prioritize: (1) adequately powered studies for rare outcomes such as mortality; (2) standardized reporting of probiotic strains, dosages, and timing; (3) longer follow-up periods; (4) cost-effectiveness analyses; and (5) identification of patient subgroups most likely to benefit. Additionally, there is an urgent need for high-quality systematic reviews that adhere to rigorous methodological standards, including pre-registration of protocols, comprehensive risk of bias assessment, and transparent reporting of funding sources.

## Supplementary Information

Additional file 1.

## Data Availability

The datasets are available from the corresponding author on reasonable request.
