# Supplementary material for: Efficacy of probiotic and synbiotic supplementation on the length of hospital stays and risk of postoperative mortality in patients undergoing surgery: an umbrella review of systematic reviews and meta-analyses of randomized clinical trials
Source: Eur J Med Res. 2026 Jan 8;31:235. doi: 10.1186/s40001-025-03756-0 (PMC12882427; doi:10.1186/s40001-025-03756-0)
Supplement: Supplementary file 1 — Additional file 1. [file 40001_2025_3756_MOESM1_ESM.docx]

| **Content** | **Page** |
| --- | --- |
| Supplementary Table 1 | 2-4 |
| Supplementary Table 2 | 5 |
| Supplementary Table 3 | 6 |
| Supplementary Table 4 | 8-9 |
| Supplementary Table 5 | 10 |
| Supplementary Table 6 | 11-12 |
| Supplementary Table 7 | 13-14 |
| Supplementary references | 15-16 |

**Supplementary Table 1.** PRIOR checklist.

| **Section**  Topic | | **#** | | **Item** | | **Location reported** | |
| --- | --- | --- | --- | --- | --- | --- | --- |
| **TITLE** | | | | | |  | |
| Title | | 1 | | Identify the report as an overview of reviews. | | Page 1 | |
| **ABSTRACT** | | | | | |  | |
| Abstract | | 2 | | Provide a comprehensive and accurate summary of the purpose, methods, and results of the overview of reviews. | | Page 2 | |
| **INTRODUCTION** | | | | | |  | |
| Rationale | | 3 | | Describe the rationale for conducting the overview of reviews in the context of existing knowledge. | | Page 4 | |
| Objectives | | 4 | | Provide an explicit statement of the objective(s) or question(s) addressed by the overview of reviews. | | Page 5 | |
| **METHODS** | | | | | |  | |
| Eligibility criteria | | 5a | | Specify the inclusion and exclusion criteria for the overview of reviews. If supplemental primary studies were included, this should be stated, with a rationale. | | Page 6 | |
|  |  | 5b | | Specify the definition of ‘systematic review’ as used in the inclusion criteria for the overview of reviews. | | Page 6 | |
| Information sources | | 6 | | Specify all databases, registers, websites, organizations, reference lists, and other sources searched or consulted to identify systematic reviews and supplemental primary studies (if included).  Specify the date when each source was last searched or consulted. | | Page 5 | |
| Search strategy | | 7 | | Present the full search strategies for all databases, registers and websites, such that they could be reproduced. Describe any search filters and limits applied. | | Table S2 | |
| Selection process | | 8a | | Describe the methods used to decide whether a systematic review or supplemental primary study (if included) met the inclusion criteria of the overview of reviews. | | Page 5 | |
|  |  | 8b | | Describe how overlap in the populations, interventions, comparators, and/or outcomes of systematic reviews was identified and managed during study selection. | | Table 1 | |
| Data collection process | | 9a | | Describe the methods used to collect data from reports. | | Page 6 | |
|  |  | 9b | | If applicable, describe the methods used to identify and manage primary study overlap at the level  of the comparison and outcome during data collection. For each outcome, specify the method used to illustrate and/or quantify the degree of primary study overlap across systematic reviews. | | Page 7 | |
|  |  | 9c | | If applicable, specify the methods used to manage discrepant data across systematic reviews during data collection. | | Page 6 | |
| Data items | | 10 | | List and define all variables and outcomes for which data were sought. Describe any assumptions made and/or measures taken to identify and clarify missing or unclear information. | | Page 6 | |
| Risk of bias assessment | | 11a | | Describe the methods used to *assess* risk of bias or methodological quality of the included systematic reviews. | | Page 7 | |
|  |  | 11b | | Describe the methods used to *collect* data on (from the systematic reviews) and/or *assess* the risk of bias of the primary studies included in the systematic reviews. Provide a justification for instances where flawed, incomplete, or missing assessments are identified but not re-assessed. | | Page 7 | |
|  |  | 11c | | Describe the methods used to *assess* the risk of bias of supplemental primary studies (if included). | | Page 7 | |
| Synthesis methods | | 12a | | Describe the methods used to summarize or synthesize results and provide a rationale for the choice(s). | | Page 7 | |
|  |  | 12b | | Describe any methods used to explore possible causes of heterogeneity among results. | | Page 8 | |
|  |  | 12c | | Describe any sensitivity analyses conducted to assess the robustness of the synthesized results. | | Not applicable | |
| Reporting bias assessment | | 13 | | Describe the methods used to *collect* data on (from the systematic reviews) and/or *assess* the risk of bias due to missing results in a summary or synthesis (arising from reporting biases at the levels of the systematic reviews, primary studies, and supplemental primary studies, if included). | | Page 7 | |
| Certainty assessment | | 14 | | Describe the methods used to *collect* data on (from the systematic reviews) and/or *assess* certainty (or confidence) in the body of evidence for an outcome. | | Page 7 | |
| **RESULTS** | | | | | |  | |
| Systematic review and supplemental primary study selection | | 15a | | Describe the results of the search and selection process, including the number of records screened, assessed for eligibility, and included in the overview of reviews, ideally with a flow diagram. | | Page 8 | |
|  |  | 15b | | Provide a list of studies that might appear to meet the inclusion criteria, but were excluded, with the main reason for exclusion. | | Table S3 | |
| **Section**  Topic | | **#** | | **Item** | | **Location reported** | |
| Characteristics of systematic reviews and supplemental primary studies | | 16 | | Cite each included systematic review and supplemental primary study (if included) and present its characteristics. | | Page 8 | |
| Primary study overlap | | 17 | | Describe the extent of primary study overlap across the included systematic reviews. | | Page 8 | |
| Risk of bias in systematic reviews, primary studies, and  supplemental primary studies | | 18a | | Present assessments of risk of bias or methodological quality for each included systematic review. | | Table S4 | |
|  |  | 18b | | Present assessments (*collected* from systematic reviews or *assessed* anew) of the risk of bias of the primary studies included in the systematic reviews. | | Table S4 | |
|  |  | 18c | | Present assessments of the risk of bias of supplemental primary studies (if included). | | Table S4 | |
| Summary or synthesis of results | | 19a | | For all outcomes, summarize the evidence from the systematic reviews and supplemental primary studies (if included). If meta-analyses were done, present for each the summary estimate and its precision and measures of statistical heterogeneity. If comparing groups, describe the direction of the effect. | | Pages 8-11 | |
|  |  | 19b | | If meta-analyses were done, present results of all investigations of possible causes of heterogeneity. | | Pages 8-11 | |
|  |  | 19c | | If meta-analyses were done, present results of all sensitivity analyses conducted to assess the robustness of synthesized results. | | Not applicable | |
| Reporting biases | | 20 | | Present assessments (*collected* from systematic reviews and/or *assessed* anew) of the risk of bias due to missing primary studies, analyses, or results in a summary or synthesis (arising from reporting biases at the levels of the systematic reviews, primary studies, and supplemental primary  studies, if included) for each summary or synthesis assessed. | | Table S4 | |
| Certainty of evidence | | 21 | | Present assessments (*collected* or *assessed* anew) of certainty (or confidence) in the body of evidence for each outcome. | | Pages 11-12 | |
| **DISCUSSION** | | | | | |  | |
| Discussion | | 22a | | Summarize the main findings, including any discrepancies in findings across the included systematic reviews and supplemental primary studies (if included). | | Page 12 | |
|  |  | 22b | | Provide a general interpretation of the results in the context of other evidence. | | Pages 12-15 | |
|  |  | 22c | | Discuss any limitations of the evidence from systematic reviews, their primary studies, and supplemental primary studies (if included) included in the overview of reviews. Discuss any limitations of the overview of reviews methods used. | | Page 15 | |
|  |  | 22d | | Discuss implications for practice, policy, and future research (both systematic reviews and primary research). Consider the relevance of the findings to the end users of the overview of reviews, e.g., healthcare providers, policymakers, patients, among others. | | Pages 15-16 | |
| **OTHER INFORMATION** | | | | | |  | |
| Registration and protocol | | 23a | | Provide registration information for the overview of reviews, including register name and registration number, or state that the overview of reviews was not registered. | | Page 5 | |
|  |  | 23b | | Indicate where the overview of reviews protocol can be accessed, or state that a protocol was not prepared. | | Page 5 | |
|  |  | 23c | | Describe and explain any amendments to information provided at registration or in the protocol. Indicate the stage of the overview of reviews at which amendments were made. | | Page 5 | |
| Support | | 24 | | Describe sources of financial or non-financial support for the overview of reviews, and the role of the funders or sponsors in the overview of reviews. | | Page 16 | |
| Competing interests | | 25 | | Declare any competing interests of the overview of reviews' authors. | | Page 16 | |
| Author Information | | 26a | | Provide contact information for the corresponding author. | | Page 1 (title page) | |
|  |  | 26b | | Describe the contributions of individual authors and identify the guarantor of the overview of reviews. | | Page 16 | |
| Availability of data and other materials | | 27 | | Report which of the following are available, where they can be found, and under which conditions they may be accessed: template data collection forms; data collected from included systematic reviews and supplemental primary studies; analytic code; any other materials used in the overview of reviews. | | Page 16 | |

| **Supplementary Table 2.** Search strategy including the key terms and the queries for databases (July 20, 2025). | |
| --- | --- |
| PubMed (n=126) | #1 : "Meta-Analysis"[Title/Abstract] OR "meta-analyses"[Title/Abstract] OR "Meta-Analysis"[Title/Abstract] OR "meta-analyze"[Title/Abstract] OR "Systematic Review"[Title/Abstract] OR "Systematic Reviews as Topic"[MeSH Terms] OR "Meta-Analysis as Topic"[MeSH Terms]  #2: "Synbiotics"[MeSH Terms] OR "Synbiotics"[Title/Abstract] OR ("Probiotics"[MeSH Terms] OR "Probiotics"[Title/Abstract] OR "Probiotic"[Title/Abstract]) OR ("Prebiotics"[MeSH Terms] OR "Prebiotics"[Title/Abstract] OR "Prebiotic"[Title/Abstract])  #3 : "General Surgery"[MeSH Terms] OR "General Surgery"[Title/Abstract] OR "Surgery"[Title/Abstract] OR "operation"[Title/Abstract]  #4: 1 AND 2 AND 3 |
| Web of Sciences  (n= 41) | #1: Meta-Analysis (Topic) or meta-analyses (Topic) or Systematic Review (Topic) or Systematic  Reviews (Topic)  #2: General Surgery (Topic) or Surgery (Topic) or operation (Topic)  #3: Synbiotics (Topic) or Probiotics (Topic) or Probiotic (Topic) or Prebiotics (Topic) or Prebiotic  (Topic)  #4:  #1 AND #2 AND #3 |
| Scopus (n=331) | #1: TITLE-ABS-KEY ( "Meta-Analysis" ) OR TITLE-ABS-KEY ( "meta-analyses" ) OR TITLE-ABS-KEY ( "Systematic Review" ) OR TITLE-ABS-KEY ( "Systematic Reviews" )  #2: TITLE-ABS-KEY ( "General Surgery" ) OR TITLE-ABS-KEY ( "Surgery" ) OR TITLE-ABS-KEY ( "operation" )  #3: TITLE-ABS-KEY ( "symbiotic" ) OR TITLE-ABS-KEY ( "Probiotics" ) OR TITLE-ABS-KEY ( "Probiotic" ) OR TITLE-ABS-KEY ( "probiotics" ) OR TITLE-ABS-KEY ( "probiotic" )  #4:  #1 AND #2 AND #3 |
| EMASE (n=237) | #1: ('meta analysis'/exp OR 'meta-analysis':ti,ab OR 'meta-analyses':ti,ab OR 'systematic review'/exp OR 'systematic review':ti,ab OR 'systematic reviews':ti,ab)  #2: ('general surgery'/exp OR 'general surgery':ti,ab OR 'surgery':ti,ab OR 'operation':ti,ab)  #3: ('synbiotic agent'/exp OR 'synbiotics':ti,ab OR 'probiotic agent'/exp OR 'probiotic':ti,ab OR 'probiotics':ti,ab OR 'prebiotic agent'/exp OR 'prebiotic':ti,ab OR 'prebiotics':ti,ab)  #1 AND #2 AND #3 |
| Cochrane liberally (n=22) | #1: ("General Surgery"):ti,ab,kw OR ("Surgery"):ti,ab,kw OR ("operation"):ti,ab,kw  #2: ("symbiotic"):ti,ab,kw OR ("Probiotics"):ti,ab,kw OR ("Probiotic"):ti,ab,kw OR ("probiotics"):ti,ab,kw OR ("probiotic"):ti,ab,kw  #3: ("Meta-Analysis"):ti,ab,kw OR ("meta-analyses"):ti,ab,kw OR ("Systematic Review"):ti,ab,kw OR ("Systematic Reviews"):ti,ab,kw  #1 AND #2 AND #3 |

**Supplementary Table 3.** Excluded studies with reason (N=28).

| 1. Prevention of postoperative recurrence of Crohn's disease 2. Perioperative Nutritional Support: A Review of Current Literature 3. Gut Microbiota in Patients with Morbid Obesity Before and After Bariatric Surgery: a Ten-Year Review Study (2009–2019) 4. Probiotics in bariatric surgery ensure greater lipids and glycemic profile with no effect on anthropometric measurements and inflammatory markers: A systematic review and meta-analysis of RCT | Different study design |
| --- | --- |
| 1. Probiotics Can Further Reduce Waist Circumference in Adults with Morbid Obesity after Bariatric Surgery: A Systematic Review and Meta-Analysis of Randomized Controlled Trials 2. Role of Probiotics in Modulating Human Gut Microbiota Populations and Activities in Patients with Colorectal Cancer—A Systematic Review of Clinical Trials 3. Effects of probiotics in patients with morbid obesity undergoing bariatric surgery: a systematic review and meta-analysis 4. Use of pro-/synbiotics as prophylaxis in patients undergoing colorectal resection for cancer: A meta-analysis of randomized controlled trials 5. Efficacy of probiotics in patients with morbid obesity undergoing bariatric surgery: a systematic review and meta-analysis 6. Prevention and management of recurrent postoperative Hirschsprung's disease obstructive symptoms and enterocolitis: Systematic review and meta-analysis 7. Probiotics for the prevention of surgical necrotising enterocolitis: systematic review and meta-analysis 8. The Effect of Perioperative Administration of Probiotics on Colorectal Cancer Surgery Outcomes 9. A review and meta-analysis of the efficacy of antibiotics and probiotics in management of pouchitis‏ 10. On the Benefit of Probiotics in the Management of Pouchitis in Patients Underwent Ileal Pouch Anal Anastomosis:A Meta-analysis of Controlled Clinical Trials 11. Meta-analysis: targeting the intestinal microbiota in prophylaxis for post-operative Crohn’s disease 12. Effects of probiotic, prebiotic or synbiotic supplementation after Roux-en-Y gastric bypass: a systematic review with meta-analysis 13. Probiotics and Synbiotics Decrease Postoperative Sepsis in Elective Gastrointestinal Surgical Patients: a Meta-Analysis 14. Probiotics used for postoperative infections in patients undergoing colorectal cancer surgery 15. Probiotics improve postoperative adaptive immunity in colorectal cancer patients: A systematic review and meta-analysis 16. Probiotics and synbiotics for preventing postoperative infectious complications in colorectal cancer patients: a systematic review and meta-analysis 17. Probiotics as a preventive strategy for surgical infection in colorectal cancer patients: a systematic review and meta-analysis of randomized trials 18. Effect of probiotics and synbiotics on complications of wound infection after colorectal surgery: A meta‐analysis. 19. Probiotics reduce postoperative infections in patients undergoing colorectal surgery: a systematic review and meta‐analysis. 20. Probiotics for preventing postoperative infection in colorectal cancer patients: a systematic review and meta-analysis. 21. Probiotics/synbiotics to reduce infectious complications after colorectal surgery: a systematic review and meta-analysis of randomised controlled trials. 22. Exploring the effect of microecological agents on postoperative immune function in patients undergoing liver cancer surgery: a systematic review and meta-analysis. 23. Comparative effectiveness of oral antibiotics, probiotics, prebiotics, and synbiotics in the prevention of postoperative infections in patients undergoing colorectal surgery: A network meta‐analysis. 24. The effect of probiotics supplementation on cancer-treatment complications: a critical umbrella review of interventional meta-analyses | Not interested outcome |
| 1. Effects of Microecological Preparations on Obese Patients after Bariatric Surgery: A Systematic Review and Meta-Analysis 2. Bowel preparation for elective procedures in children: a systematic review and meta-analysis 3. Do patients fed enterally post–gastrointestinal surgery experience more complications when fed a fiber-enriched feed compared with a standard feed? A systematic review 4. Effectiveness of prehabilitation modalities on postoperative outcomes following colorectal cancer surgery: a systematic review of randomised controlled trials. 5. Pre-Operative Immunonutrition Enhances Postoperative Outcomes and Elevates Tumor-Infiltrating Lymphocyte Counts in Colorectal Cancer   Patients: A Meta-Analysis of Randomized Controlled Trials | Not interested intervention |
| 1. Facilitating early recovery of bowel motility after colorectal surgery: a systematic review 2. The effects of microbiome-targeted therapy on cognitive impairment and postoperative cognitive dysfunction — A systematic review 3. Surgical Prehabilitation in Patients with Gastrointestinal Cancers: Impact of Unimodal and Multimodal Programs on Postoperative Outcomes and Prospects for New Therapeutic Strategies—A Systematic Review 4. Postoperative changes of the microbiome: are surgical complications related to the gut flora? A systematic review 5. Microbiome modulation in the prevention and management of colorectal cancer: a systematic review of clinical interventions 6. Probiotics supplementation in patients with colorectal cancer: a systematic review of randomized controlled trials 7. The Effects of Probiotics on Reducing the Colorectal Cancer Surgery Complications: A Periodic Review during 2007-2017 8. Gut Microbiota, Probiotics and Psychological States and Behaviors after Bariatric Surgery—A Systematic Review of Their Interrelation | Systematic review without meta-analysis |

| **Supplementary Table 4**: Overlap of primary studies across included systematic reviews and meta-analyses | | | |
| --- | --- | --- | --- |
| **Primary RCT Study** | **Population/Surgery Type** | **Outcome Reported** | **Meta-Analyses Including This Study** |
| Anderson et al., 2004 | Elective surgical patients | Hospital stay | Lytvyn, 2016; Yang, 2017; Skonieczna-Żydecka, 2018; An, 2022; Chowdhury, 2020; Kinross, 2013 |
| Bajramagic et al., 2019 | Colorectal cancer | Mortality | Amitay, 2020; An, 2022; Araujo, 2023; Chen, 2024; Chen, 2022; Liu, 2017 |
| Chen et al., 2014 | Colorectal cancer | Hospital stay | An, 2022; Araujo, 2023; Chen, 2016; Chen, 2022; Liu, 2017; Veziant, 2022 |
| Costeloe et al., 2016 | Preterm infants | Mortality | Trivedi, 2024 |
| Eguchi et al., 2011 | Liver transplantation | Hospital stay, ICU stay | Lytvyn, 2016; Kahn, 2020; Sawas, 2015; Wu, 2018 |
| Folwarski et al., 2021 | Pancreatoduodenectomy | Mortality | Takagi, 2019; Tang, 2021 |
| Franko et al., 2019 | Major abdominal surgery | Hospital stay, Mortality | Lytvyn, 2016; Chowdhury, 2020; Wu, 2018 |
| Grąt et al., 2017 | Liver transplantation | Hospital stay | Kahn, 2020; Sawas, 2015 |
| Iida et al., 2020 | Hepatic resection | Hospital stay | Gan, 2019; Kahn, 2020 |
| Kakaei et al., 2019 | Colorectal cancer | Hospital stay | An, 2022; Araujo, 2023; Chen, 2022 |
| Kanazawa et al., 2005 | Biliary cancer, Hepatectomy | Hospital stay, ICU stay | Gan, 2019; Kahn, 2020; Tang, 2022; Sugawara, 2006 |
| Lages et al., 2018 | Head and neck cancer | Hospital stay | Single study inclusion |
| Liu et al., 2011 | Colorectal cancer | Hospital stay, Mortality | Amitay, 2020; An, 2022; Araujo, 2023; Chen, 2024; Chen, 2016; Chen, 2022; Liu, 2017; Veziant, 2022; Yang, 2016 |
| Liu et al., 2015 | Colorectal liver metastases | Hospital stay | An, 2022; Araujo, 2023; Gan, 2019; Kahn, 2020 |
| Liu et al., 2022 | Gastric cancer | Hospital stay | Ye, 2023 |
| Liu et al., 2023 | Gastric cancer | Hospital stay | Ye, 2023 |
| Mallick et al., 2022 | Living donor liver transplant | Hospital stay, ICU stay | Kahn, 2020; Sawas, 2015 |
| Mangell et al., 2012 | Colon resection | Mortality | Amitay, 2020; An, 2022 |
| Manzoni et al., 2006 | Preterm neonates | Mortality | Trivedi, 2024 |
| McNaught et al., 2002 | Elective surgical patients | Mortality | Lytvyn, 2016; Chowdhury, 2020; Kinross, 2013 |
| Mizuta et al., 2016 | Colorectal surgery | Hospital stay | An, 2022; Araujo, 2023; Chen, 2022 |
| Nomura et al., 2007 | Pancreatoduodenectomy | Mortality | Takagi, 2019; Tang, 2021 |
| Pellino et al., 2013 | Colorectal surgery | Hospital stay | An, 2022; Araujo, 2023; Chen, 2022 |
| Polakowski et al., 2019 | Colorectal cancer | Hospital stay | An, 2022; Araujo, 2023; Chen, 2022 |
| Rammohan et al., 2015 | Chronic pancreatitis | ICU stay | Takagi, 2019 |
| Rao et al., 2022 | Congenital GI surgery | Hospital stay | Trivedi, 2024 |
| Rayes et al., 2002a | Major abdominal surgery | Hospital stay | Lytvyn, 2016; Chowdhury, 2020; Kinross, 2013 |
| Rayes et al., 2002b | Liver transplantation | Hospital stay, ICU stay | Kahn, 2020; Sawas, 2015 |
| Rayes et al., 2005 | Liver transplantation | Hospital stay, Mortality | Kahn, 2020; Sawas, 2015 |
| Rayes et al., 2007 | Pancreatoduodenectomy | Hospital stay, ICU stay, Mortality | Takagi, 2019; Tang, 2021 |
| Russolillo et al., 2014 | HPB surgery | Mortality | Gan, 2019; Tang, 2022 |
| Sari et al., 2011 | Very low birth weight infants | Mortality | Trivedi, 2024 |
| Shen et al., 2022 | Colorectal cancer | Hospital stay | An, 2022; Araujo, 2023 |
| Sommacal et al., 2015 | Periampullary neoplasms | Hospital stay, Mortality | Takagi, 2019; Tang, 2021 |
| Sugawara et al., 2006 | Biliary cancer surgery | Hospital stay, Mortality | Gan, 2019; Tang, 2022 |
| Tan et al., 2016 | Colorectal cancer | Hospital stay | An, 2022; Araujo, 2023; Chen, 2022 |
| Tanaka et al., 2012 | Esophageal cancer | Hospital stay, ICU stay | Tang, 2022 |
| Usami et al., 2011 | Hepatic surgery | Hospital stay, ICU stay | Gan, 2019; Kahn, 2020 |
| Xie et al., 2018 | Gastric cancer | Hospital stay | Ye, 2023 |
| Xu et al., 2020 | Gastric cancer | Hospital stay | Ye, 2023 |
| Yang et al., 2016 | Colorectal cancer | Hospital stay | Amitay, 2020; An, 2022; Araujo, 2023; Chen, 2024; Chen, 2022; Liu, 2017; Veziant, 2022 |
| Yokoyama et al., 2014 | Esophagectomy | Hospital stay, Mortality | Tang, 2022 |
| Yokoyama et al., 2016 | Pancreatoduodenectomy | Hospital stay, Mortality | Takagi, 2019; Tang, 2021 |
| Yoshiya et al., 2025 | Living-donor liver transplant | Hospital stay | Kahn, 2020 |
| Zhang et al., 2012 | Colorectal cancer | Hospital stay | An, 2022; Araujo, 2023; Chen, 2022 |
| Zhang et al., 2013 | Liver transplantation | Hospital stay | Kahn, 2020; Sawas, 2015 |
| Zhao et al., 2017 | Gastric cancer | Hospital stay | Ye, 2023 |

**Supplementary Table 5.** GRADE evidence table for efficacy of probiotics and synbiotics supplementation on length of hospital stay and risk of postoperative mortality in patients undergoing surgery

| **Certainty assessment** | | | | | | | **№ of patients** | | **Effect** | | **Certainty** | **Importance** |
| --- | --- | --- | --- | --- | --- | --- | --- | --- | --- | --- | --- | --- |
| **№ of studies** | **Study design** | **Risk of bias** | **Inconsistency** | **Indirectness** | **Imprecision** | **Other considerations** | **Intervention** | **placebo** | **Relative (95% CI)** | **Absolute (95% CI)** |  |  |
| **Probiotic** | | | | | | | | | | | | |
| **Length of hospital stay** | | | | | | | | | | | | |
| 16 | randomized trials | not serious | serious | not serious | not serious | not serious | 796 | 801 | MD, -1.00 (-1.37, -0.64) | **-** | ⊕⊕⊕◯  Medium | IMPORTANT |
| **Risk of postoperative mortality** | | | | | | | | | | | | |
| 11 | randomized trials | not serious | not serious | not serious | serious | not serious | 1171 | 1190 | RR, 0.82 (0.57, 1.19) | RR, -0.24 (-0.62, 0.15) | ⊕⊕⊕◯  Medium | IMPORTANT |
| **Synbiotic**  **Length of hospital stay** | | | | | | | | | | | | |
| 16 | randomized trials | not serious | serious | not serious | not serious | not serious | 610 | 748 | MD, -2.57 (-4.51, -0.64) | - | ⊕⊕⊕◯  Medium | IMPORTANT |

**Length of ICU stay**

| 8 | randomized trials | not serious | serious | not serious | serious | not serious | 301 | 302 | MD, -0.42 (-1.00, 0.16) | - | ⊕⊕◯◯  Low | IMPORTANT |
| --- | --- | --- | --- | --- | --- | --- | --- | --- | --- | --- | --- | --- |

**Risk of postoperative mortality**

| 8 | randomized trials | not serious | not serious | not serious | serious | not serious | 259 | 253 | RR, 0.64 (0.33, 1.38) | RR, 0.10 (-0.27, 0.47) | ⊕⊕⊕◯  Medium | IMPORTANT |
| --- | --- | --- | --- | --- | --- | --- | --- | --- | --- | --- | --- | --- |

**Supplementary Table 6.** Adverse events following probiotics or symbiotic in primary trials included in the umbrella review.

|  | **Probiotics** |
| --- | --- |
| **Reported adverse events** | **Author, year** |
| Preoperatively, with five patients experiencing shortfalls due to reasons such as taste (2), nausea (1). Postoperatively, the median consumption was 800 ml over 5 days, with common issues being taste (19) and nausea (16). | McNaught et al, 2002 |
| Not reported | Nomura et al, 2007 |
| Not reported | Liu et al, 2011 |
| No adverse effects were recorded after the administration of high doses of Lactobacillus plantarum 299v. | Mangell et al, 2012 |
| Specifically, in the study, 2 out of 34 patients in the probiotic group experienced diarrhea, and 3 out of 34 patients had abdominal cramps. In the control group that received only fiber, 1 out of 33 patients experienced diarrhea, and 6 out of 33 patients had abdominal distension and cramps. | Zhang et al, 2012 |
| This suggests that diarrhea is a noted side effect in the study, with a significantly lower incidence in the group treated with probiotics compared to the control group. | Liu et al, 2015 |
| Not reported | Mizuta et al, 2015 |
| It was found that while the incidence of diarrhea was significantly lower in the probiotics group compared to the placebo group, other gastrointestinal side effects such as nausea, vomiting, and abdominal cramps can still occur but were not statistically different between the two groups. | Yang et al, 2015 |
| Not reported | Tan et al, 2016 |
| Not reported | Chen et al, 2014 |
| Five out of eleven patients readmitted in the probiotic group were readmitted due to dehydration resulting from diet intolerance and/or diarrhea. | Franko et al, 2019 |
| Not reported | Folwarski et al, 2021 |
| The test group experienced 8 cases of diarrhea and 8 cases of vomiting, whereas the control group experienced 18 cases of diarrhea and 10 cases of vomiting, with a significantly lower incidence of diarrhea in the test group (p=0.03) but no significant difference in vomiting (p=0.61). | Xie et al, 2018 |
| Not reported | Xu et al, 2020 |
| Not reported | Liu et al, 2022 |
| No patients were excluded or dropped out in present study. | Liu et al, 2023 |
| No adverse effects to probiotic group or placebo were observed. | Pellino et al, 2013 |
| No probiotic-associated adverse events were reported. | Costeloe et al, 2015 |
| No adverse effects potentially associated with the probiotic were recorded. | Manzoni et al, 2006 |
| No probiotic-associated adverse events were reported. | Sari et al, 2011 |
| Not reported | Zhao et al, 2017 |
| Not reported | Bajramagic et al, 2019 |
| Adverse events were similar in both groups: six in the probiotics group (four diarrhea, one nausea, and one constipation) and six in the control group (three diarrhea, two nausea, and one constipation). | Grat et al, 2017 |
| Not reported | Rao et al, 2022 |
| Not reported | Shen et al, 2022 |
| Not reported | Kakaei et al, 2019 |
|  | **Synbiotic** |
| **Reported adverse events** | **Author, year** |
| Not reported | Kanazawa et al, 2005 |
| Not reported | Sugawara et al, 2006 |
| without any adverse effect | Usami et al, 2011 |
| Specifically, three patients in the symbiotic group had transient diarrhea, and one patient experienced nausea​. | Russolillo et al, 2014 |
| Not reported | Sommacal et al, 2015 |
| Not reported | Polakowski et al, 2019 |
| Four patients in the symbiotic group experienced diarrhea, which was related to the intake of oligofructose, the prebiotic component of the therapy. One patient in the symbiotic group found the oligofructose preparation unpalatable. | Anderson et al, 2004 |
| Flatulence: Reported by 5 patients in both the symbiotic and placebo groups. Abdominal bloating: Reported by 1 patient in the symbiotic group and 3 patients in the placebo group. Abdominal cramps: Reported by 2 patients in both groups. | Lages et al, 2018 |
| The intervention group experienced mild side effects: 6 cases of abdominal distention and 4 cases of abdominal cramps. No severe diarrhea or vomiting occurred. | Rayes et al, 2002 |
| The study noted that abdominal side effects such as distension, cramps, or diarrhea were observed. Specifically, these side effects were seen in 8 of 32 patients in the SBD group, 6 of 31 patients in the live Lactobacillus group, and 11 of 32 patients in the inactivated Lactobacillus group. | Rayes-2 et al, 2002 |
| Two out of 40 patients in intervention group experienced diarrhea, and 3 out of 40 patients had abdominal cramps. In contrast, in control group, 2 out of 40 patients experienced diarrhea, and 6 out of 40 patients had abdominal cramps and abdominal distension. | Rayes et al, 2007 |
| In the group receiving the symbiotic combination (group A), 3 out of 33 patients developed diarrhea and 5 out of 33 patients experienced abdominal cramps. Similarly, in the group receiving only fibers (group B), 4 out of 33 patients showed signs of diarrhea and 6 out of 33 patients had abdominal distension and cramps. | Rayes et al, 2005 |
| Not reported | Yokoyama et al, 2014 |
| Not reported | Yokoyama et al, 2016 |
| Not reported | Eguchi et al, 2011 |
| Not reported | Rammohan et al, 2015 |
| No adverse events occurred because of symbiotic administration. | Tanaka et al, 2012 |
| No serious side effects related to symbiotics have been observed. In group A (symbiotic + EN), 2 out of 34 patients developed diarrhea and 3 out of 34 patients abdominal cramps; and in group B(only fiber + EN), 1 out of 33 patients had signs of diarrhea and 6 out of 33 patients abdominal distension and cramps. All side effects disappeared under temporary reduction in the amount of enteral nutrition. | Zhang et al, 2013 |
| Eight in the intervention group (three diarrhea, three pain abdomen, and two bloating) and nine in the control group (three diarrhea, four pain abdomen, and two bloating). | Mallick et al, 2022 |
| Not reported | Lida et al, 2020 |
| No adverse events occurred because of symbiotic administration. | Yoshiya et al, 2025 |

| **Supplementary Table 7.**  Methodological quality of included systematic reviews using AMSTAR 2. | | | | | | | | | | | | | | | | | |
| --- | --- | --- | --- | --- | --- | --- | --- | --- | --- | --- | --- | --- | --- | --- | --- | --- | --- |
| **Author, year (ref.)** | **Q1** | **Q2** | **Q3** | **Q4** | **Q5** | **Q6** | **Q7** | **Q8** | **Q9** | **Q10** | **Q11** | **Q12** | **Q13** | **Q14** | **Q15** | **Q16** | **Quality of evidence** |
| An et al, 2022 [1] | **Y** | **Y** | **Y** | **Y** | **Y** | **Y** | **Y** | **Y** | **Y** | **N** | **Y** | **Y** | **Y** | **Y** | **Y** | **Y** | **Low** |
| Araujo et al, 2022 [2] | **Y** | **Y** | **Y** | **Y** | **Y** | **Y** | **Y** | **Y** | **Y** | **N** | **Y** | **Y** | **Y** | **Y** | **Y** | **Y** | **Low** |
| Arumugam et al, 2016 [3] | **Y** | **N** | **Y** | **Y** | **N** | **N** | **Y** | **Y** | **Y** | **N** | **Y** | **N** | **N** | **Y** | **Y** | **N** | **Critically Low** |
| Amitay et al., 2020 [4] | **Y** | **Y** | **Y** | **Y** | **Y** | **Y** | **Y** | **Y** | **Y** | **Y** | **Y** | **Y** | **Y** | **Y** | **PY** | **Y** | **High** |
| Cogo et al., 2021 [5] | **Y** | **Y** | **Y** | **Y** | **Y** | **Y** | **Y** | **Y** | **Y** | **Y** | **Y** | **Y** | **Y** | **Y** | **N** | **Y** | **Critically low** |
| Chen et al., 2023 [6] | **Y** | **Y** | **Y** | **Y** | **Y** | **Y** | **Y** | **Y** | **Y** | **N** | **Y** | **Y** | **Y** | **Y** | **Y** | **Y** | **Low** |
| Chen et al., 2016 [7] | **Y** | **Y** | **Y** | **Y** | **Y** | **Y** | **Y** | **Y** | **Y** | **N** | **Y** | **Y** | **Y** | **Y** | **Y** | **Y** | **Low** |
| Chowdhury et al, 2020 [8] | **Y** | **Y** | **Y** | **Y** | **Y** | **Y** | **Y** | **Y** | **Y** | **PY** | **Y** | **Y** | **Y** | **Y** | **Y** | **Y** | **High** |
| Gan et al., 2019 [9] | **Y** | **N** | **Y** | **Y** | **Y** | **Y** | **Y** | **Y** | **Y** | **N** | **Y** | **Y** | **Y** | **Y** | **Y** | **Y** | **Low** |
| Kahn et al, 2020 [10] | **Y** | **Y** | **Y** | **Y** | **Y** | **Y** | **Y** | **Y** | **Y** | **N** | **Y** | **Y** | **Y** | **Y** | **Y** | **Y** | **Low** |
| Kinross et al., 2012 [11] | **Y** | **N** | **Y** | **Y** | **Y** | **Y** | **Y** | **Y** | **Y** | **N** | **Y** | **Y** | **Y** | **Y** | **Y** | **Y** | **Critically low** |
| Lytvyn et al, 2016 [12] | **Y** | **Y** | **Y** | **Y** | **Y** | **Y** | **Y** | **Y** | **Y** | **N** | **Y** | **Y** | **Y** | **Y** | **Y** | **Y** | **Low** |
| Pitsouni et al., 2009 [13] | **Y** | **N** | **Y** | **Y** | **Y** | **Y** | **Y** | **Y** | **Y** | **N** | **Y** | **Y** | **Y** | **Y** | **N** | **Y** | **Critically low** |
| Persson et al, 2024 [14] | **PY** | **Y** | **Y** | **Y** | **Y** | **Y** | **N** | **PY** | **Y** | **N** | **Y** | **Y** | **Y** | **Y** | **Y** | **Y** | **Low** |
| Rees et al., 2017 [15] | **Y** | **PY** | **Y** | **Y** | **Y** | **Y** | **Y** | **Y** | **Y** | **Y** | **Y** | **Y** | **Y** | **Y** | **Y** | **N** | **High** |
| Sawas et al., 2015 [16] | **Y** | **N** | **Y** | **Y** | **Y** | **Y** | **Y** | **Y** | **Y** | **N** | **Y** | **Y** | **Y** | **Y** | **N** | **Y** | **Critically low** |
| Skonieczna-Żydecka et al., 2018 [17] | **Y** | **N** | **Y** | **Y** | **Y** | **Y** | **Y** | **Y** | **Y** | **N** | **Y** | **Y** | **Y** | **Y** | **Y** | **Y** | **Critically low** |
| Tang et al, 2022 [18] | **Y** | **N** | **Y** | **Y** | **Y** | **Y** | **Y** | **Y** | **Y** | **N** | **Y** | **Y** | **Y** | **Y** | **Y** | **Y** | **Critically low** |
| Tang et al, 2022-1 [19] | **Y** | **N** | **Y** | **Y** | **Y** | **Y** | **Y** | **Y** | **Y** | **Y** | **Y** | **Y** | **N** | **Y** | **Y** | **Y** | **Critically low** |
| Tang et al, 2021 [20] | **Y** | **Y** | **Y** | **Y** | **Y** | **Y** | **Y** | **Y** | **Y** | **N** | **Y** | **Y** | **Y** | **Y** | **Y** | **Y** | **Low** |
| Takagi et al, 2019 [21] | **Y** | **N** | **Y** | **Y** | **Y** | **Y** | **Y** | **Y** | **Y** | **N** | **Y** | **Y** | **Y** | **Y** | **N** | **Y** | **Critically low** |
| Wu et al, 2018-1 [22] | **Y** | **N** | **Y** | **Y** | **Y** | **Y** | **Y** | **Y** | **Y** | **N** | **Y** | **Y** | **Y** | **Y** | **Y** | **Y** | **Critically low** |
| Wu et al, 2018 [23] | **Y** | **Y** | **Y** | **Y** | **Y** | **Y** | **Y** | **Y** | **Y** | **N** | **Y** | **Y** | **Y** | **Y** | **Y** | **Y** | **Low** |
| Yang et al, 2016 [24] | **Y** | **N** | **Y** | **Y** | **Y** | **Y** | **Y** | **Y** | **Y** | **N** | **Y** | **Y** | **Y** | **Y** | **Y** | **Y** | **Critically low** |
| Ye et al, 2023 [25] | **Y** | **Y** | **Y** | **Y** | **Y** | **Y** | **Y** | **Y** | **Y** | **N** | **Y** | **Y** | **Y** | **Y** | **Y** | **Y** | **Low** |
| Zeng et al, 2021 [26] | **Y** | **Y** | **Y** | **Y** | **Y** | **Y** | **Y** | **Y** | **Y** | **N** | **Y** | **Y** | **Y** | **Y** | **Y** | **Y** | **Low** |
| Karitnig et al, 2025 [27] | **Y** | **Y** | **Y** | **Y** | **Y** | **Y** | **Y** | **Y** | **Y** | **Y** | **Y** | **Y** | **Y** | **Y** | **N** | **Y** | **Low** |
| Paterson et al, 2025 [28] | Y | Y | Y | Y | Y | Y | Y | Y | Y | Y | Y | Y | Y | Y | Y | Y | High |
| Trivedi et al, 2024 [29] | **Y** | **Y** | **Y** | **Y** | **Y** | **Y** | **Y** | **Y** | **Y** | **Y** | **NA** | **Y** | **Y** | **Y** | **NA** | **Y** | **High** |
| Wu et al, 2025 [30] | **Y** | **Y** | **Y** | **Y** | **Y** | **Y** | **Y** | **Y** | **Y** | **Y** | **Y** | **Y** | **Y** | **Y** | **Y** | **Y** | **High** |

ref, reference; PY, partially yes. Q1: Did the research questions and inclusion criteria for the review include the components of PICO?, Q2: 2. Did the report of the review contain an explicit statement that the review methods were established prior to the conduct of the review and did the report justify any significant deviations from the protocol?; Q3, Did the review authors explain their selection of the study designs for inclusion in the review?; Q4, Did the review authors use a comprehensive literature search strategy?; Q5, Did the review authors perform study selection in duplicate?; Q6, Did the review authors perform data extraction in duplicate?; Q7, Did the review authors provide a list of excluded studies and justify the exclusions?; Q8, Did the review authors describe the included studies in adequate detail?; Q9, Did the review authors use a satisfactory technique for assessing the risk of bias?; Q10, Did the review authors report on the sources of funding?; Q11, Did the review authors use appropriate methods for statistical combination of results?; Q12, Did the review authors assess the potential impact of RoB in individual studies on the results?; Q13, Did the review authors account for RoB in individual studies when interpreting/ discussing the results of the review?; Q14, Did the review authors provide a satisfactory explanation for, and discussion of, any heterogeneity?; Q15, Did the review authors carry out an adequate investigation of publication bias?; Q16, Did the review authors report any potential sources of conflict of interest?

**References:**

1. An, S., et al., *Perioperative probiotics application for preventing postoperative complications in patients with colorectal cancer: A systematic review and meta-analysis.* Medicina, 2022. **58**(11): p. 1644.

2. Araujo, M.M., et al., *The effect of probiotics on postsurgical complications in patients with colorectal cancer: a systematic review and meta-analysis.* Nutrition Reviews, 2023. **81**(5): p. 493-510.

3. Arumugam, S., C.S. Lau, and R.S. Chamberlain, *Probiotics and synbiotics decrease postoperative sepsis in elective gastrointestinal surgical patients: a meta-analysis.* Journal of Gastrointestinal Surgery, 2016. **20**(6): p. 1123-1131.

4. Amitay, E.L., et al., *Probiotic/synbiotic treatment and postoperative complications in colorectal cancer patients: systematic review and meta-analysis of randomized controlled trials.* Clinical and Translational Gastroenterology, 2020. **11**(12): p. e00268.

5. Cogo, E., et al., *Probiotics evaluation in oncological surgery: A systematic review of 36 randomized controlled trials assessing 21 diverse formulations.* Current Oncology, 2021. **28**(6): p. 5192-5214.

6. Chen, J., et al., *Efficacy and safety of oral probiotic supplementation in mitigating postoperative surgical site infections in patients undergoing colorectal cancer surgery: A systematic review and meta‐analysis.* International Wound Journal, 2024. **21**(4): p. e14603.

7. Chen, W., et al., *Perioperative pro-/synbiotic for colorectal surgery: a systematic review and updated meta-analysis of randomized controlled trials.* Int. J. Clin. Exp. Med, 2016. **9**(6): p. 9681-93.

8. Chowdhury, A.H., et al., *Perioperative probiotics or synbiotics in adults undergoing elective abdominal surgery: a systematic review and meta-analysis of randomized controlled trials*. 2020, LWW. p. 1036-1047.

9. Gan, Y., et al., *Efficacy of Probiotics and Prebiotics in Prevention of Infectious Complications Following Hepatic Resections: Systematic Review and Meta-Analysis.* Journal of Gastrointestinal & Liver Diseases, 2019. **28**(2).

10. Kahn, J., G. Pregartner, and P. Schemmer, *Effects of both pro-and synbiotics in liver surgery and transplantation with special focus on the gut–liver axis—a systematic review and meta-analysis.* Nutrients, 2020. **12**(8): p. 2461.

11. Kinross, J.M., et al., *A meta‐analysis of probiotic and synbiotic use in elective surgery: does nutrition modulation of the gut microbiome improve clinical outcome?* Journal of Parenteral and Enteral Nutrition, 2013. **37**(2): p. 243-253.

12. Lytvyn, L., et al., *Probiotics and synbiotics for the prevention of postoperative infections following abdominal surgery: a systematic review and meta-analysis of randomized controlled trials.* Journal of hospital infection, 2016. **92**(2): p. 130-139.

13. Pitsouni, E., et al., *Does the use of probiotics/synbiotics prevent postoperative infections in patients undergoing abdominal surgery? A meta-analysis of randomized controlled trials.* European journal of clinical pharmacology, 2009. **65**: p. 561-570.

14. Persson, J.E., et al., *Perioperative or Postoperative Probiotics Reduce Treatment-Related Complications in Adult Colorectal Cancer Patients Undergoing Surgery: A Systematic Review and Meta-analysis.* Journal of Gastrointestinal Cancer, 2024: p. 1-9.

15. Rees, C.M., et al., *Probiotics for the prevention of surgical necrotising enterocolitis: systematic review and meta-analysis.* BMJ paediatrics open, 2017. **1**(1).

16. Sawas, T., et al., *Patients receiving prebiotics and probiotics before liver transplantation develop fewer infections than controls: a systematic review and meta-analysis.* Clinical Gastroenterology and Hepatology, 2015. **13**(9): p. 1567-1574. e3.

17. Skonieczna-Żydecka, K., et al., *A systematic review, meta-analysis, and meta-regression evaluating the efficacy and mechanisms of action of probiotics and synbiotics in the prevention of surgical site infections and surgery-related complications.* Journal of clinical Medicine, 2018. **7**(12): p. 556.

18. Tang, G., et al., *Prophylactic effects of probiotics or synbiotics on postoperative ileus after gastrointestinal cancer surgery: A meta-analysis of randomized controlled trials.* PLoS One, 2022. **17**(3): p. e0264759.

19. Tang, G., et al., *Probiotics or synbiotics for preventing postoperative infection in hepatopancreatobiliary cancer patients: a meta-analysis of randomized controlled trials.* Nutrition and Cancer, 2022. **74**(10): p. 3468-3478.

20. Tang, G., et al., *Effects of perioperative probiotics and synbiotics on pancreaticoduodenectomy patients: a meta-analysis of randomized controlled trials.* Frontiers in Nutrition, 2021. **8**: p. 715788.

21. Takagi, K., et al., *Current evidence of nutritional therapy in pancreatoduodenectomy: Systematic review of randomized controlled trials.* Annals of gastroenterological surgery, 2019. **3**(6): p. 620-629.

22. Wu, X.D., et al., *Efficacy of prophylactic probiotics in combination with antibiotics versus antibiotics alone for colorectal surgery: A meta‐analysis of randomized controlled trials.* Journal of Surgical Oncology, 2018. **117**(7): p. 1394-1404.

23. Wu, X.-D., et al., *Effects of perioperative supplementation with pro-/synbiotics on clinical outcomes in surgical patients: a meta-analysis with trial sequential analysis of randomized controlled trials.* Clinical Nutrition, 2018. **37**(2): p. 505-515.

24. Yang, Z., et al., *Effect of perioperative probiotics and synbiotics on postoperative infections after gastrointestinal surgery: a systematic review with meta‐analysis.* Journal of Parenteral and Enteral Nutrition, 2017. **41**(6): p. 1051-1062.

25. Ye, W., et al., *The effect of probiotics on surgical outcomes in patients with gastric cancer: a meta-analysis of randomized controlled trials.* Frontiers in Surgery, 2023. **10**: p. 1254597.

26. Zeng, J., et al., *The effect of pro/synbiotics on postoperative infections in colorectal cancer patients: A systematic review and meta-analysis.* Complementary Therapies in Clinical Practice, 2021. **43**: p. 101370.

27. Karitnig, R., et al., *Value of Probiotics on Outcome in Patients Following Liver Surgery: A Systematic Review and Meta-Analysis.* Medicina, 2025. **61**(6): p. 1068.

28. Paterson, C., et al., *Do Perioperative Probiotics/Synbiotics Reduce Postoperative Infection Rates Following Elective Colorectal Surgery? A Systematic Review and Meta-Analysis.* Journal of Surgical Research, 2025. **312**: p. 163-176.

29. Trivedi, A., E. Teo, and K.S. Walker, *Probiotics for the postoperative management of term neonates after gastrointestinal surgery.* Cochrane Database of Systematic Reviews, 2024(1).

30. Wu, H., et al., *The effect of perioperative probiotics and synbiotics on postoperative infections in patients undergoing major liver surgery: a meta-analysis of randomized controlled trials.* PeerJ, 2025. **13**: p. e18874.
